# Recent advances in self‐powered and flexible UVC photodetectors

**DOI:** 10.1002/EXP.20210078

**Published:** 2022-05-25

**Authors:** Thi My Huyen Nguyen, Seong Gwan Shin, Hyung Wook Choi, Chung Wung Bark

**Affiliations:** ^1^ Department of Electrical Engineering Gachon University Seongnam Gyeonggi Republic of Korea

**Keywords:** photo‐absorber, photoconductor, responsivity, UV radiation

## Abstract

Ultraviolet‐C (UVC) radiation is employed in various applications, including irreplaceable applications in military and civil fields, such as missile guidance, flame detection, partial discharge detection, disinfection, and wireless communication. Although most modern electronics are based on Si, UVC detection technology remains a unique exception because the short wavelength of UV radiation makes efficient detection with Si difficult. In this review, recent challenges in obtaining ideal UVC photodetectors with various materials and various forms are introduced. An ideal photodetector must satisfy the following requirements: high sensitivity, fast response speed, high on/off photocurrent ratio, good regional selectivity, outstanding reproducibility, and superior thermal and photo stabilities. UVC detection is still in its infancy compared to the detection of UVA as well as other photon spectra, and recent research has focused on different key components, including the configuration, material, and substrate, to acquire battery‐free, super‐sensitive, ultra‐stable, ultra‐small, and portable UVC photodetectors. We introduce and discuss the strategies for fabricating self‐powered UVC photodetectors on flexible substrates in terms of the structure, material, and direction of incoming radiation. We also explain the physical mechanisms of self‐powered devices with various architectures. Finally, we present a brief outlook that discusses the challenges and future strategies for deep‐UVC photodetectors.

## Graphical abstract

This review introduced and discussed strategies for fabricating self‐powered UVC photodetectors in terms of the device structure, photoactive material, and flexible substrates. Based on the summary of previous works, we presented a brief outlook of challenges and future strategies for deep‐UVC photodetectors.

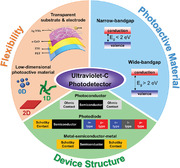

## 1 INTRODUCTION

**FIGURE 1:**
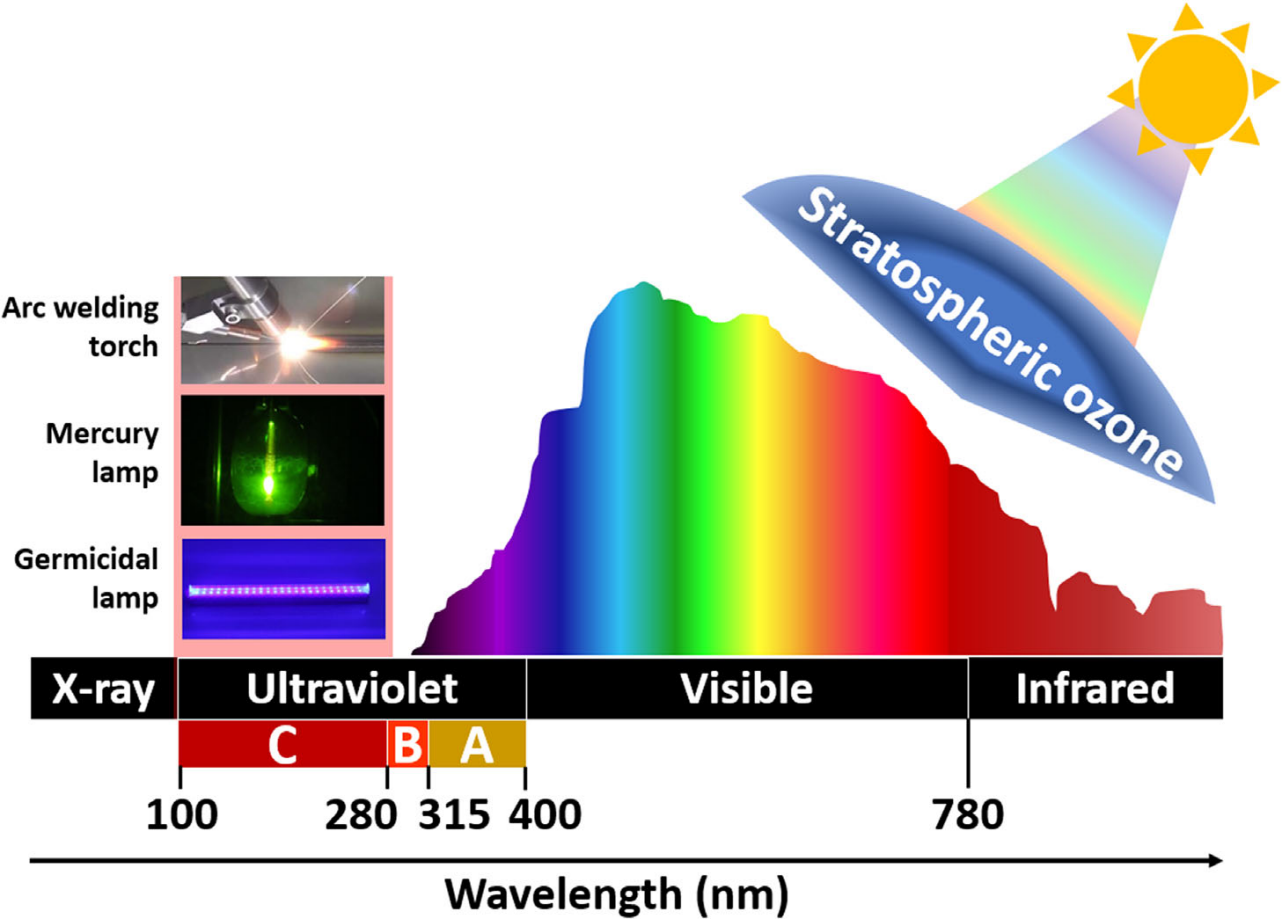
Classification of UV light by wavelength

Ultraviolet (UV) radiation is an ordinary component of solar radiation. Although it accounts for less than 10% of the total sunlight, UV radiation has a severe influence on humanity. The International Commission on Illumination (CIE) divides the spectrum of solar electromagnetic radiation into three bands, as shown in Figure 1: UVA (315–400 nm), UVB (280–315 nm), and UVC (100–280 nm).^[^ ^1^ ^]^ Among them, UVA and UVB are mainly derived from the natural environment. They account for 90%–95% and 5%–10% of solar radiation, respectively, and are of the part of sunlight that leads to sunburn, skin cancers, and cataracts.^[^ ^2^, ^3^, ^4^, ^5^ ^]^

Until now, many photodetectors, which detect UV radiation with wavelengths longer than 280 nm, have been integrated into weather forecast programs to prevent UVA and UVB overexposure.^[^ ^6^ ^]^ In contrast, the deep‐UVC band, which is also called the solar‐blind region and is completely absorbed by the atmospheric ozone layer, requires further research to provide alerts for overexposure and to harness its power. UVC rays are emitted from artificial sources, such as arc welding torches, mercury lamps, germicidal lamps, and UV sanitizing bulbs or sunbeds.

It is noteworthy that unlike UVA and UVB, UVC causes serious lesions, even with extremely low exposure.^[^ ^7^, ^8^, ^9^, ^10^ ^]^ For instance, Trevisan et al.^[^ ^11^ ^]^ reported that acute exposure to short wavelengths (245–290 nm) can induce damage at the DNA level in mammalian cells. Despite these hazards, UVC radiation is applied in various irreplaceable applications in the military and civil fields, such as missile guidance, flame detection, partial discharge detection, disinfection, and wireless communication.^[^ ^12^, ^13^, ^14^, ^15^ ^]^ Consequently, UVC detectors have received tremendous research attention. Suitable photodetectors can be employed to detect UVC overexposure or monitor UV emissions from high‐voltage systems, depending on their design.

An ideal UVC photodetector must satisfy the following requirements: high sensitivity, fast response speed, high on/off photocurrent ratio, good regional selectivity, outstanding reproducibility, and superior thermal and photo stabilities.^[^ ^16^, ^17^ ^]^ However, a practical UVC photodetector must also be self‐powered and flexible.

Regarding self‐power, most conventional photodetectors require input power, which makes the system complex and expensive. To solve this problem, there has been substantial research on UV detectors that operate without an external power source, namely self‐powered photodetectors. Unlike the complex constituents and high‐voltage operation of photomultiplier tubes, the working principles of self‐powered UVC photodetectors are based on the photovoltaic effect of various photoactive materials (also called photo‐absorbers) along with special device structures. On the one hand, photoactive materials possessing narrow bandgaps, such as silicon and halide perovskites, are favorable for transferring photocarriers to electrodes, leading to a high current signal. On the other hand, intrinsically wide‐bandgap photo‐absorbers with limited structural defect states are expected to exhibit high selectivity in the UVC region with a minimal current signal in other regions (e.g., UVA and visible spectra).

Regarding flexibility, traditional UVC photodetectors were built on rigid quartz substrates that suffer from numerous drawbacks, including high cost, fragility, strict preparation, and rigorous operation. Therefore, flexible UVC photodetectors can be more widely applied and have outstanding advantages. Flexible devices can be utilized in wearable and portable electronics rather than rigid devices because they are small and lightweight, and their traits allow the device to bend, roll, or stretch. Common flexible substrates for photodetectors include polyethylene naphthalate (PEN),^[^ ^18^ ^]^ polyethylene terephthalate (PET),^[^ ^19^, ^20^ ^]^ polystyrene (PS),^[^ ^21^ ^]^ and polyimide (PI).^[^ ^22^ ^]^ Interestingly, they exhibit a maximum transmittance at a wavelength of 300 nm. Thus, UVC photodetectors must be grown in an appropriate configuration. For this reason, the effect of the direction of incoming UVC radiation is presented in this review.

In this review, we introduce flexible UVC photodetectors based on diverse functional semiconductors, representing UVC absorber materials. Furthermore, we explain the physical mechanisms of self‐powered devices with various architectures. After that, we discuss different types of flexible substrates along with the corresponding light illumination directions. Finally, we present a brief outlook that discusses the challenges and upcoming strategies for deep‐UVC photodetectors.

## 2 KEY PARAMETERS FOR EVALUATING THE EFFICIENCY OF UVC PHOTODETECTORS

Various parameters can be used to characterize UV photodetectors, and the important parameters are summarized below.

1. Dark current (*I*_dark_): The dark current is the small current created in a device when there is no incident light. The value of the dark current should be minimized for a good detector. The dark current mainly originates from the leakage of electrons from one electrode to the opposite one. Additionally, the dark current is a measure of the recombination in the device.^[^ ^23^ ^]^ Diodes with large recombinations have large dark currents. Normally, the dark current injection can be reduced by using a thin charge‐blocking layer with a high lowest unoccupied molecular orbital level and low electron mobility, as reported previously, and by the reduction of extrinsic defects and trap states in the photoactive layer.
2. Photocurrent (*I*_ph_): The photocurrent is the large amount of current generated by the photogenerated carriers under irradiation. When light is incident on the detector, it provides an energy greater than the bandgap, and electrons in the valence band can rise to the conduction band, increasing the amount of current in the conduction band. The intensity of the photocurrent is related to the light intensity and bias voltage.
3. Sensitivity: The sensitivity is the ability of the device to detect a weak signal from a UV light source. It is defined as the ratio of the photocurrent (*I*_ph_) to the dark current (*I*_dark_).
(1) $$\mathrm{Sensivity}=\frac{I_{\mathrm{ph}}}{I_{\mathrm{dark}}}$$
4. Responsivity (*R*): The responsivity is defined as the output photocurrent or photovoltage divided by the input light power on the active area of the photodetector, and the units are A W^−1^ or V W^−1^. It represents the efficient response of the photodetector to the light signal and can be expressed as
(2) $$R=\frac{I_{\mathrm{ph}}\;or\;V_{\mathrm{ph}}}{P_{\mathrm{in}}}$$

where photocurrent *I*_ph _= *I*_light_ − *I*_dark_ (or photovoltage *V*_ph _= *V*_light_ − *V*_dark_), and *P*_in_ is the incident power.

5 Response speed: The response speed reflects how quickly the photodetector responds to the optical signal and is characterized by the rise and fall times. The rise time is the time required for the response to increase from 10% to 90% of its maximum value, and the fall time is the time required for the response to decrease from 90% to 10% of its maximum value. The response speed of the photodetector is affected by the trapping effect, capacitive effect, and saturation rate of the semiconductor carrier.
6 Specific detectivity (D*): The detectivity is a key performance index for the comparison of photodetectors with different materials and shapes. It indicates the minimum level of optical power that the photodetector can detect. The detectivity is independent of the area because the noise on the device is proportional to the square root of the area. In noise‐dominated devices, D* can be written as
(3) $$D^{*}=\frac{R}{\sqrt{2qJ_{\mathrm{dark}}}}$$

where D* is measured in cm Hz^1/2^ W^−1^ (Jones). *R* is the responsivity (A W^−1^), *q* is the charge of an electron (C), and *J*_dark_ is the dark current density (A cm^−2^).

7 External quantum efficiency (EQE): The EQE is the ratio of the number of charge carriers collected by the photodetector to the number of incident photons, indicating the conversion efficiency of the photodetector. The EQE of a photodetector can be expressed as a spectral response by
(4) $$\mathrm{EQE}=\frac{Rhc}{q\lambda }$$

where *R* is the measured responsivity, *q* is the elementary electron charge, *λ* is the wavelength of the incident light, *h* is Planck's constant, and *c* is the speed of light.

8 Flexibility and stability: The flexibility and stability of a photodetector can be determined by measuring the performance (e.g., dark current and photocurrent) under various mechanical stresses, such as bending angle/radius, or continuous physical impact conditions, such as multiple bending/unbending cycles.

## 3 CLASSIFICATION OF UVC PHOTODETECTORS

Photomultiplier tubes (PMTs) and semiconductor‐based devices are the two most prevalent types of UVC photodetectors. Although photodetectors have been traditionally made from PMTs, devices based on semiconductors have been more attractive recently. In this regard, a large number of narrow‐bandgap and wide‐bandgap materials have been employed as UVC‐absorbing photoactive layers. They have both merits and shortcomings, which are detailed below.

### 3.1 Photomultiplier tubes

A typical PMT configuration consists of a photocathode, a diode, and an anode. The principle of operation is that when light is irradiated to the photocathode, electrons are emitted owing to the photoelectric effect. The electrons are amplified by passing through the diode; then, they pass through the anode to generate an electrical signal in an external circuit. PMTs can detect low‐intensity light because the signal is amplified without an external device. As such, PMTs have been widely used as optical sensors for scintillation detectors because of their large detection area and high sensitivity. However, PMT‐based detectors require vacuum‐pressure conditions and a large bias voltage (>100 V) for operation, which results in heavy systems with high power consumption. They can also be disturbed by external magnetic fields, further restricting their use. Therefore, new strategies are required to develop integrated devices by miniaturizing the components for portability and eliminating the external high‐voltage source for flexibility.

### 3.2 Semiconductor‐based charge‐coupled devices

Apart from PMT‐based detectors, miniaturization of devices using semiconductors exhibit outstanding advantages such as lightweight, flexibility, portability, low power consumption, or even self‐powered operation. Therefore, researchers have deeply considered the device configuration for fabricating a self‐powered UVC photodetector. That is responsible for the conversion of the carrier‐to‐electrical signal. The detailed introduction of mechanisms based on device structures is clarified below.

#### 3.2.1 Mechanisms for self‐powered UVC photodetectors based on the structure

##### Photoconductors

**FIGURE 2:**
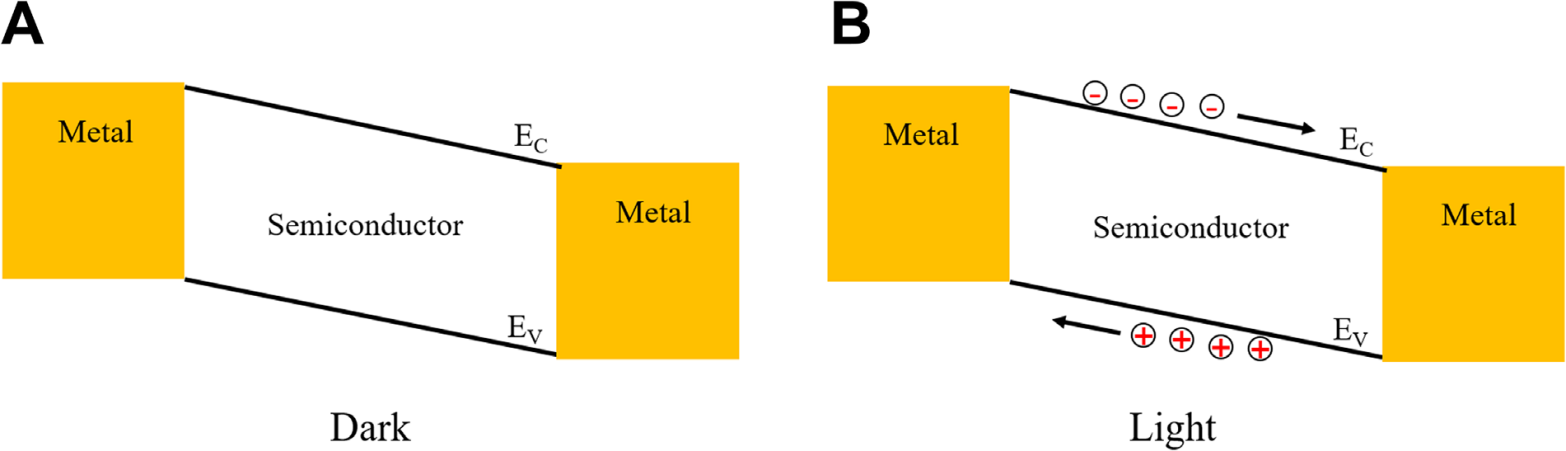
(A,B) Schematic of the energy band diagram of a photoconductor

A photoconductor is a photodetector based on the photoconductive effect of semiconductors.^[^ ^24^, ^25^ ^]^ Typically, photoconductors are made of semiconductors with metal ohmic contacts at both ends (Figure 2A). When the device is irradiated with photons of energy greater than the bandgap, electron–hole pairs are created and separated by internal and external bias, effectively increasing the conductivity of the device (Figure 2B).^[^ ^26^ ^]^

##### p–n and p–i–n photodiodes

**FIGURE 3:**
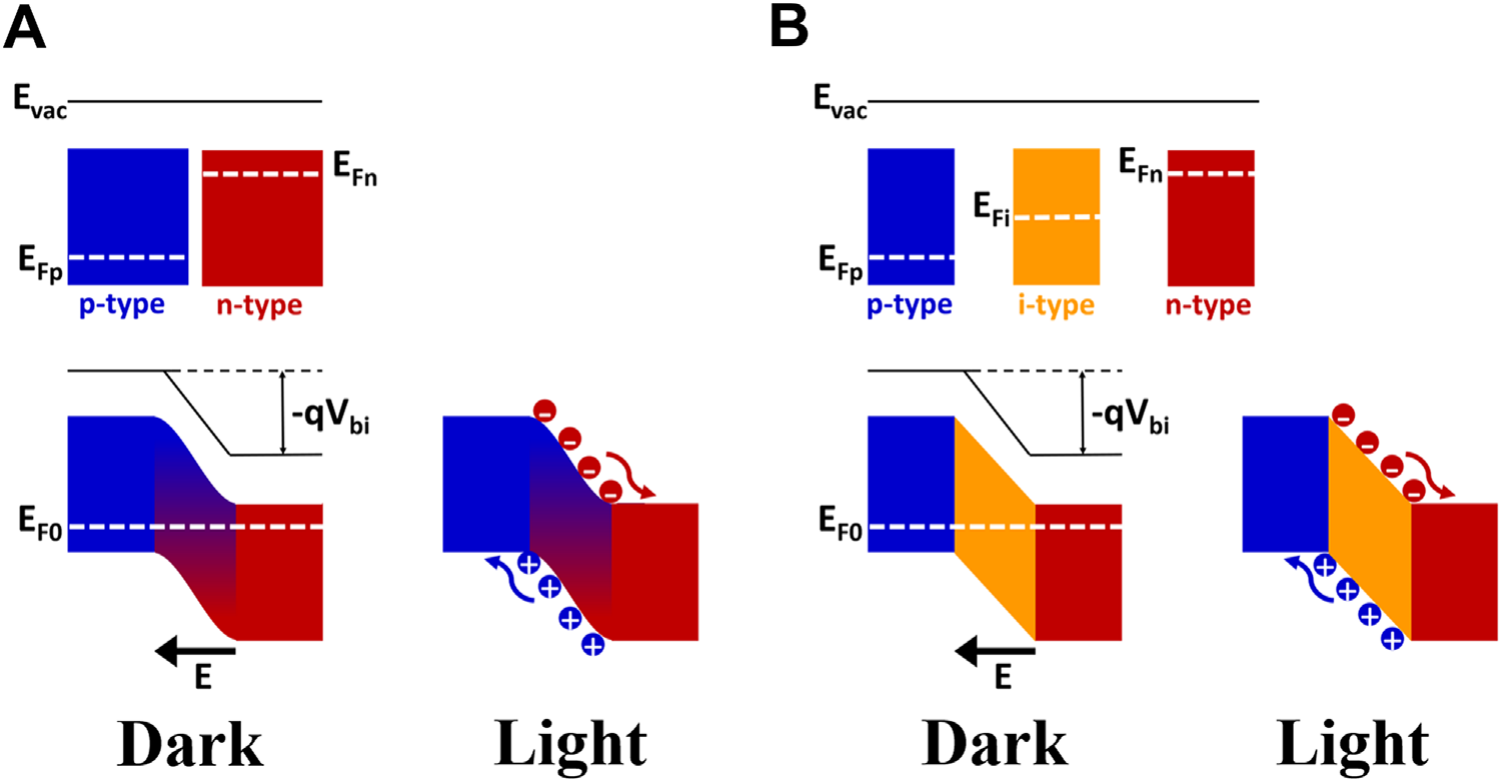
Working mechanisms of self‐powered UVC photodiodes with (A) p–n and (B) p–i–n structures

The physical mechanisms of self‐powered UVC photodetectors in p–n and p–i–n devices are illustrated in Figures 3A and B, respectively. Generally, the generated electron–hole pairs in the photon‐harvester (or photoactive region) are separated automatically owing to the built‐in electric field induced by contacting suitable constituents (i.e., p‐, i‐, and n‐type semiconductors) along with their energy bands.

The GaN/Ga_2_O_3_ heterojunction is an example of the p–n architecture, which is constructed with a well‐matched energy band.^[^ ^27^ ^]^ As shown in the mechanism schematic (Figure 3A), the charge carriers will flow until the Fermi levels align after contacting p‐ and n‐type semiconductors. A depletion region is created at the interface of the p–n junction, which is the core workspace for self‐powered photoconductors. In addition, a built‐in potential barrier, which contributes to the separation of photogenerated carriers, is established owing to the difference between the work functions of p‐type and n‐type semiconductors.

When the device is under dark conditions, the carriers from oxygen vacancy traps and holes in the materials flow through the device, which forms the dark current. When the device is illuminated under UVC light, the UVC radiation penetrates the depletion layer to generate electrons and holes. The excited charge carriers are separated by a built‐in electric field and are driven by band alignment. Consequently, the device can operate without an external power source. Moreover, the electron–hole pairs will separate faster and more effectively if the built‐in potential is larger.

Therefore, some researchers have moved the Fermi level of n‐type semiconductors close to the conduction band by using doped n‐type semiconductors instead of pure n‐type semiconductors.^[^ ^28^ ^]^ Another strategy for enhancing the photoactive area is to sandwich an intrinsic layer that is able to highly absorb deep‐UV spectra between p‐ and n‐layers as a width‐controllable depletion layer. This can remarkably improve the sensitivity and responsivity of self‐powered photoconductors.

The working principle of p–i–n photodetectors is similar to that of p–n junction devices; the intrinsic i‐layer is used as a UVC absorber to induce a large numbers of electron–hole pairs, similar to the p–n depletion layer. As shown in Figure 3B, the Fermi level equilibrates and aligns when the layers are in contact, thereby establishing a built‐in potential (V_bi_) owing to the high work function of the p‐layer and the low work function of the n‐layer. The conduction and valence bands of the intrinsic i‐layer are inclined by an electrical field, where the distance of inclination depends on the thickness of the UV absorber layer. Consequently, in this structure, the electrical field causes the drift of the photoinduced electrons and holes in the intrinsic region to the n‐ and p‐regions, respectively, in opposite directions. Subsequently, the separated electrons are quickly transferred through a high‐electron‐mobility n‐layer to one electrode, whereas holes are transferred through a high‐hole‐mobility p‐layer to the other electrode. The charge carriers are driven by matching the energy levels, resulting in a photodetector that can operate without a power supply.

##### Schottky diodes

**FIGURE 4:**
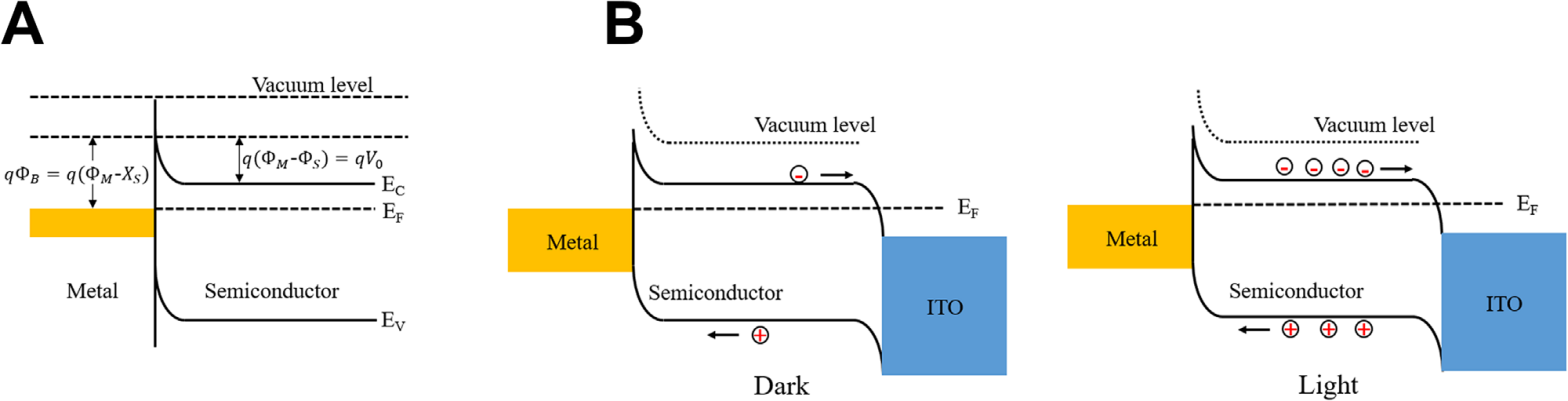
(A) Schematic of a Schottky junction. (B) Energy band diagram of an Au–MoS_2_–ITO Schottky structure under dark and light conditions

Schottky junctions are formed by metal–semiconductor contacts (Figure 4A), which form a potential barrier owing to the difference between the work functions of the metal and semiconductor.^[^ ^29^, ^30^ ^]^ The height of the potential barrier is expressed as the difference between the work function of the metal (Φ_M_) and the electron affinity of the semiconductor (*X*_s_), where *q* is the elementary charge:^[^ ^31^, ^32^ ^]^

(5) $$q\Phi_{B}=q(\Phi_{M}-X_{S}$$

When a metal and semiconductor are bonded, electrons are transferred from the semiconductor to the metal until the Fermi levels are aligned, forming a state of thermal equilibrium. Then, a potential barrier for electrons is created between the metal and semiconductor, which is called a Schottky barrier. For example, Figure 4B shows the energy bands of a Schottky junction fabricated using an Au–MoS_2_–ITO stack.^[^ ^33^ ^]^ Because the work function of metals is higher than that of semiconductors, electrons are transferred from the semiconductor to the metal to maintain the Fermi energy balance. When the device is illuminated by light, the valence band electrons of the semiconductor are excited into the conduction band and separated owing to the internal electric field, forming a photovoltage that drives an external circuit.

##### Metal–semiconductor–metal devices

**FIGURE 5:**
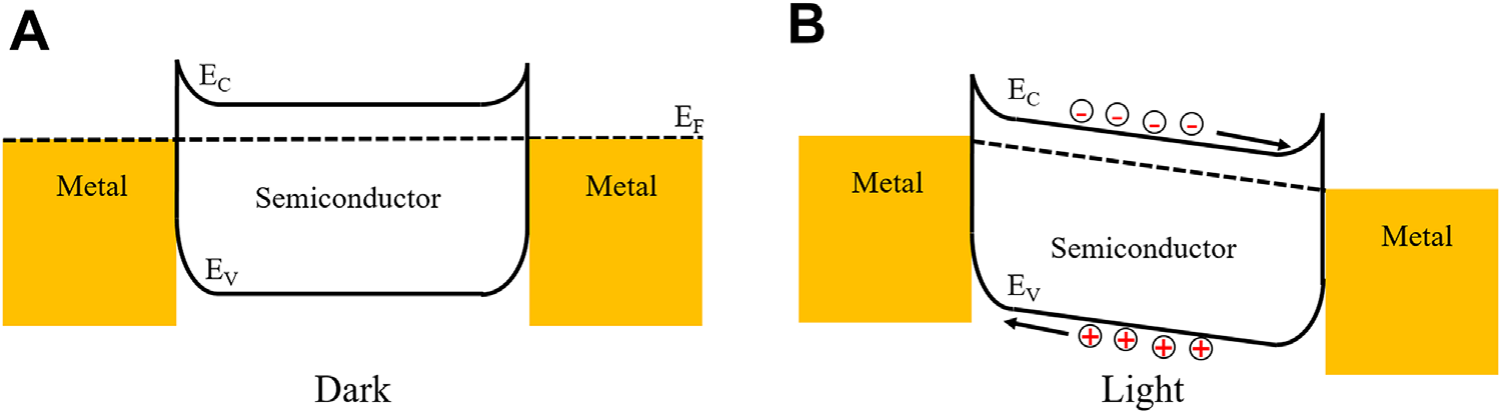
Schematic of the energy band diagram of an MSM photodetector

The MSM structure is constructed by the Schottky bonding of two electrodes to a semiconductor.^[^ ^34^, ^35^ ^]^ When an MSM device is irradiated with light, the photons create electron–hole pairs inside the semiconductor, which are transferred to opposite electrodes. The operating mechanism of MSM photodetectors is shown in Figure 5. The voltage applied to the MSM detector collapses the internal electric field and creates a new type of electric field.^[^ ^36^, ^37^ ^]^ At one junction, a reverse bias is applied, increasing the area of the space‐charge region at the semiconductor and the junction, thereby increasing the height of the potential barrier. The other junction is forward‐biased. When light is irradiated near the reverse‐biased junction, electron–hole pairs are created and transferred in opposite directions by the internal electric field. Considering photodetectors with symmetric electrodes (e.g., Au/Au, Ag/Ag at the same thickness), mirror symmetry in the Schottky barrier profile will be formed at both ends of the metal‐semiconductor contacts. In this case, the generated electrons/holes in the semiconductor layer have no priority drift direction. Therefore, an external voltage is required to support the separation of photoexcited carriers. To implement the self‐powered photodetector with MSM structure, asymmetric electrodes (e.g., Au/Ag electrode pair or Au/Au with different thicknesses) are used. The difference in barrier heights at two metal‐semiconductor interfaces causes the breaking of the mirror symmetry in the Schottky barrier profile, or in the order words, the number of carriers is different on opposite sides, leading to form a built‐in electric field. Thereby, the force of built‐in potential is responsible for the diffusion of electrons and holes. As a result, self‐power characteristic occurs in the asymmetric MSM photodetectors.^[^ ^38^, ^39^, ^40^ ^]^

#### 3.2.2 Semiconductor materials

Aside from building a low or self‐powered device with a suitable configuration, the selection of an efficient photo‐absorber layer is also an important issue. It plays for the conversion of photon‐to‐carriers that relate to the performance of a device. The photo‐absorber material using semiconductors typically is divided into narrow‐bandgap materials (bandgap E_g_ < 2 eV) and wide‐bandgap materials (bandgap E_g _> 2 eV).^[^ ^41^ ^]^ Overall, narrow‐bandgap photo‐absorbers display high carrier mobility, mature processes, and low cost. Nevertheless, the detection band is a broad range. Therefore, narrow‐bandgap UVC photodetectors need to cooperate with an optical bandpass filter to prevent interference by other spectra from sunlight. Meanwhile, wide‐bandgap photo‐absorbers exhibit intrinsic solar‐blindness and a strong radiation harness that can apply in a harsh environment. However, the carrier mobility is lower than narrow‐bandgap semiconductors.^[^ ^42^, ^43^ ^]^ The advantages and disadvantages of each semiconductor material will be further discussed below.

##### Narrow‐bandgap materials

To demonstrate the development in this field, Table 1 summarizes some important figure‐of‐merit parameters of representative UVC detectors based on different narrow‐bandgap semiconductors. UVC photodiodes based on narrow‐bandgap semiconductors commonly employ Si (bandgap of 1.12 eV). The most important features of Si‐based photodiodes are their excellent sensitivity, good linearity, and excellent UV response. In contrast, the remaining challenges are their very low selectivity to the UV spectrum (i.e., visible/infrared radiation‐blind UV detection is impossible) and the degradation in quantum efficiency in the deep‐UV range caused by the Si oxide passivation layer surrounding the Si surfaces. Moreover, a technical and economic assessment of Si‐based detectors asserted that they have sophisticated and expensive production, require ultrahigh‐temperature processing, and are expensive owing to the high cost of purified Si and UV bandpass filters.

**TABLE 1:** Performance of recent UVC photodetectors based on narrow‐bandgap materials

Photo‐absorber	Device structure	Wavelength (nm)	Voltage (V)	*R* (mA W^−1^)	*D* (Jones)	Rise/fall time	Flexible device	Ref.
Silicon photodiode (incorporated with CsPbBr_3_)	PIN (Thorlabs, FDS100)	278	5	84	7.4 × 10^12^			[44]
Silicon photodiode (incorporated with CsPbClBr_2_)	PIN	200 270	0	54 32		0.48/1.03 ms		[45]
Si_3_N_4_/Si	Heterojunction	185	0 5	4.6 325		19.3/1.8 s		[46]
CsPbBr_3_	MSM	254	2	0.24	1.9 × 10^10^	260/280 ms	Yes	[47]
MAPbI_3_	MSM	265	4	12 × 10^3^	0.1 × 10^12^	2.2/4 ms	Yes	[48]
(FAPbI_3_)_0.97_(MAPbBr_3_)_0.03_	Heterojunction	254	0	53	4.65 × 10^11^	46/47 ms		[49]
CsPbBr_3_ (incorporated with Cs_4_PbBr_6_)	PIN	254	0	49.4	1.2 × 10^12^	7.8/33.6 µs		[50]
MAPbI_3_ (incorporated with CsPbX_3_)	Heterojunction	279	0.1	1.4	2.4 × 10^11^	< 70 ms		[51]
MAPbCl_3_ MAPbl_3_ MAPbBr_3_	MSM	255	5	450 120 300		15 ms 2.5 ms 2 ms		[52]

**FIGURE 6:**
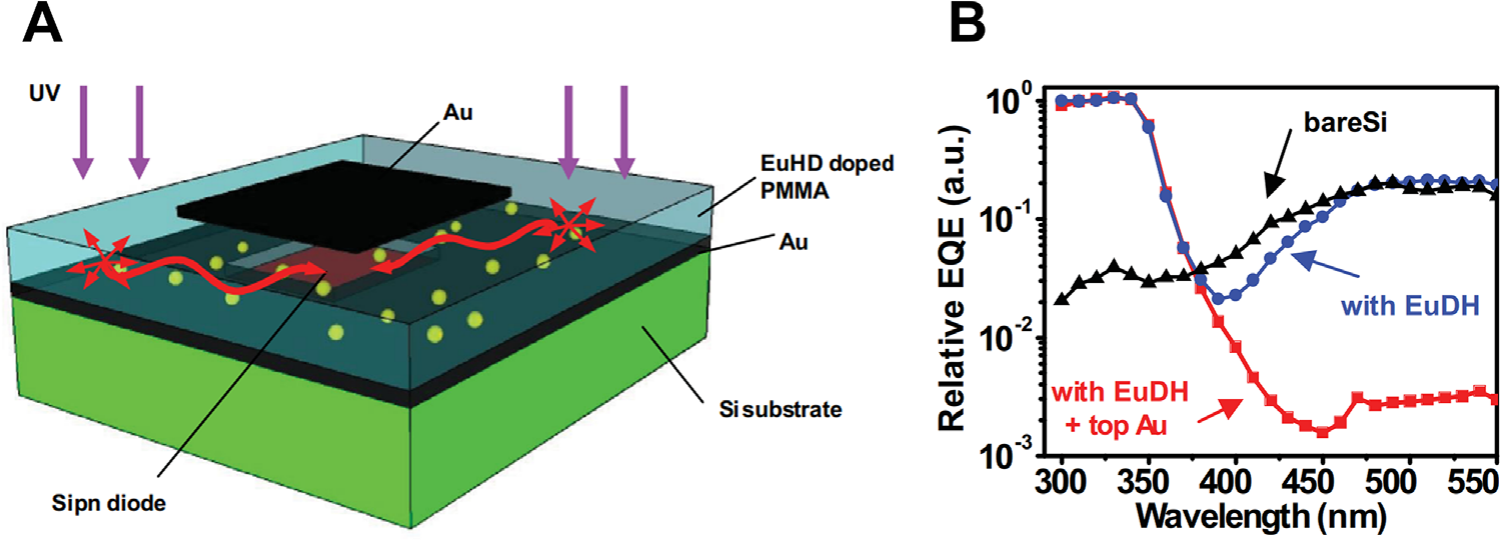
(A) Schematic of a Si photodiode using an (EuHD)‐doped PMMA film as a UV bandpass filter. (B) EQE spectra of the Si photodiode with and without the EuHD‐doped PMMA film. (A,B) Reproduced with permission.^[^ ^53^ ^]^ Copyright 2014, Wiley‐VCH

Sheng et al. combined Eu(DPEPO)(hfac)_3_ (DPEPO, bis(2‐(diphenylphosphino)phenyl)ether oxide; hfac, hexafluoroacetylacetonate) with a poly(methyl methacrylate) (PMMA) matrix to serve as a down‐conversion film for a Si photodiode, which absorbed light at 360 nm and emitted at 610 nm.^[^ ^53^ ^]^ Incorporating this UV bandpass filter remarkably enhanced the selectivity of the Si photodiode, with the rejection increasing by three orders of magnitude between 360 and 450 nm (Figure 6). Although the device detected light in the UVB region, this study confirmed that detection of the desired wavelength is feasible by using an appropriate down‐conversion filter. In other words, UVC radiation can be detected with high selectivity using Si‐based photodiodes by incorporating UVC‐to‐visible light materials dispersed into a highly transparent matrix.

In comparison, organic–inorganic hybrid perovskites and mixed‐halide perovskites have relatively simple production processes, including solution fabrication at relatively low temperatures. Low‐cost perovskite materials can be fabricated with facile procedures but can achieve high efficiency and large‐scale production. In addition to the advantageous synthesis methods, these types of perovskite materials exhibit significant potential for UVC photodetectors owing to their large optical absorption coefficient, high charge carrier mobility, tunable bandgap (1.4–2.3 eV), and low defect density.^[^ ^54^ ^]^ Therefore, UVC photodetectors using these perovskites to replace Si photodiodes have emerged with increasing frequency over the past few years.

**FIGURE 7:**
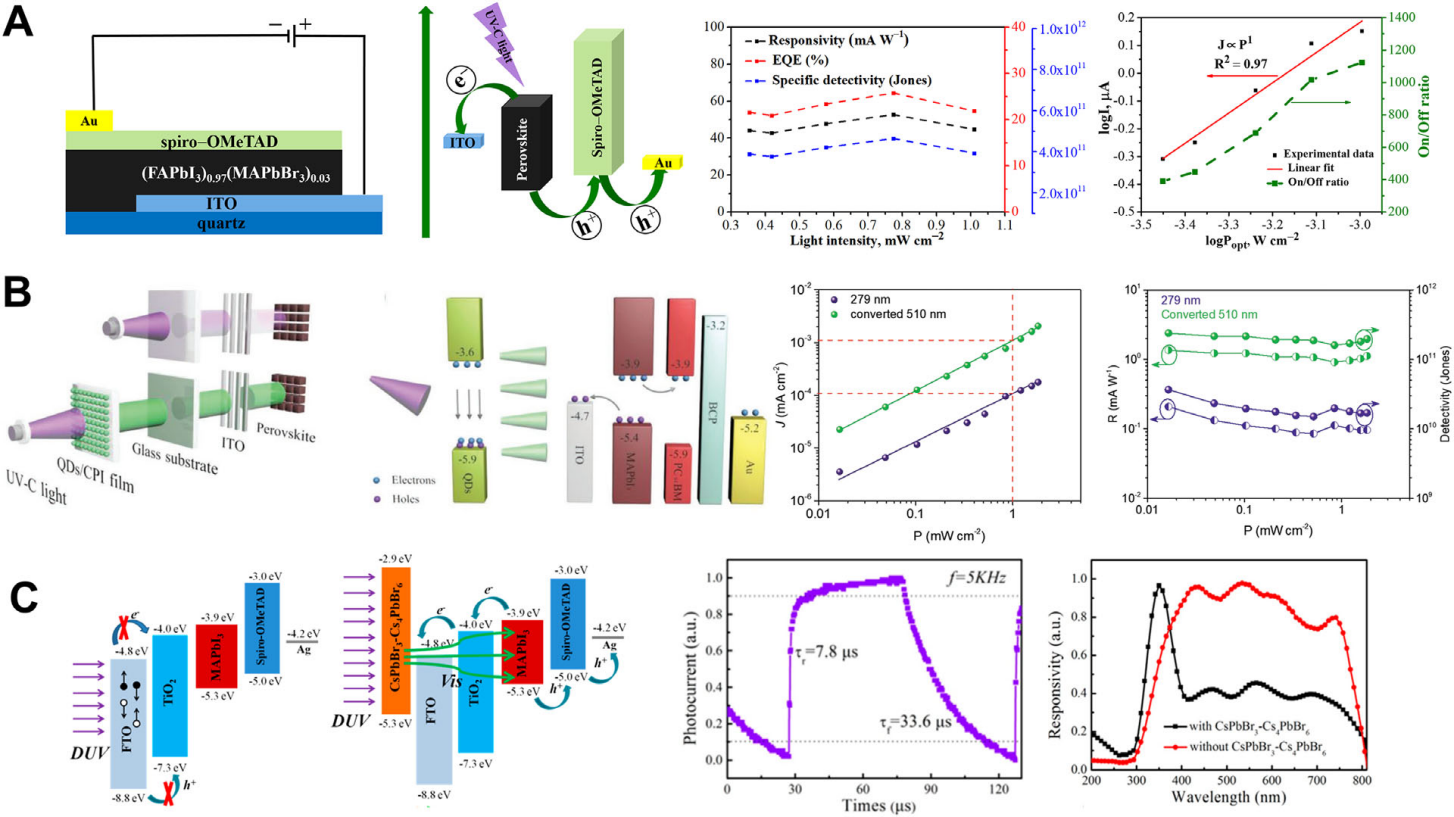
(A) Architecture and performance (responsivity, detectivity, and photocurrent) of a narrow‐bandgap mixed‐halide perovskite‐based UVC photodetector. Reproduced with permission.^[49]^ Copyright 2021, The Royal Society of Chemistry. (B) without and (C) with an integrated down‐conversion window layer. (B) Reproduced with permission.^[^ ^51^ ^]^ Copyright 2019, Wiley‐VCH. (C) Reproduced with permission.^[^ ^50^ ^]^ Copyright 2018, American Chemical Society

For instance, Zhang et al.^[^ ^52^ ^]^ developed a highly sensitive photodetector with rapid response in the 255 nm deep‐UV region using CH_3_NH_3_PbX_3_ (X = Cl, Br, I) perovskites. The responsivities were 10–10^3^ times higher than those of MgZnO and Ga_2_O_3_ detectors. Similarly, in our previous study,^[^ ^49^ ^]^ we fabricated a UVC photodetector based on (FAPbI_3_)_0.97_(MAPbBr_3_)_0.03_ via a simple solution process. As shown in Figure 7A, the material displayed excellent photoresponse under 254 nm illumination without external power, including an outstanding on/off photocurrent ratio of more than 10^3^, a good responsivity of 52.68 mA W^−1^, and a high detectivity of 4.65 × 10^10^ Jones.

Nonetheless, the use of narrow‐bandgap semiconductors for detection in the UVC region has an inherent problem: low stability. In particular, the lifespan of a narrow‐bandgap light‐harvester‐based device is shortened under prolonged exposure to extremely high photon energies (>4.4 eV).^[^ ^55^, ^56^, ^57^ ^]^ Another drawback hindering the mixed‐halide perovskites (e.g., MAPb(I_1−_ *_x_* Br*_x_*)_3_) from using as the photo‐absorber for UV detector is that the mixed‐halide perovskite undergoes the halide segregation due to exposing to UV lights.^[^ ^58^, ^59^, ^60^ ^]^ The demixing halide leads to a local reduction of the bandgap and charge‐carrier trapping, resulting in a significant loss of photocurrent.^[^ ^58^ ^]^ To overcome the halide segregation, there are two primary approaches including (i) the bromide content lower than 20% (commonly, 0.1 < *x* < 0.2);^[^ ^59^ ^]^ (ii) keeping electron/hole diffusion length less than 13 nm by the usage of mesoporous hybrid perovskite instead of conventional planar perovskite^.[^ ^60^ ^]^ Regarding an external approach, which does not affect the photo‐absorber, down‐conversion mechanisms have emerged as an alternative approach to improve the stability of narrow‐bandgap‐based photodetectors. The integration of a down‐conversion layer in perovskite‐based photodetectors can prevent significant degradation from direct exposure to deep‐UV light.

In particular, a down‐conversion window layer is inserted into the rear constituent of the detector to convert the incident deep‐UV irradiation with high‐energy photons down into longer‐wavelength regions with low‐energy photons. These photons can penetrate typical commercial ITO–glass or FTO–glass substrates to irradiate a narrow‐bandgap perovskite absorber layer. For example, Zou et al.^[^ ^51^ ^]^ fabricated a UVC photodetector based on a MAPbI_3_ perovskite, employing CsPbX_3_ quantum dots as an effective down‐conversion layer to convert direct 279 nm UV irradiation into 510 nm visible light irradiation (Figure 7B). Using a similar strategy, Tong et al.^[^ ^50^ ^]^ developed a deep‐UV photodetector with a high responsivity of 49.4 mA W^−1^ and a fast response time of 7.8/33.6 µs using a dual‐phase (CsPbBr_3_–Cs_4_PbBr_6_) inorganic perovskite as the down‐conversion layer (Figure 7C).

Recently, Mxene, which is considered a state‐of‐art class of narrow‐bandgap material, has been applied to the UVC detection field. Generally, MXenes are 2D materials consisting of M (early transitional metal such as Ti, Ta, Mo, and Cr), X (carbon or nitrogen), and T (terminal groups such as ─O, ─OH, ─F, and ─Cl). MXenes can be represented with the general formula M*_n_*_+1_X*_n_* T*_x_*, where *n* = 1, 2, 3, and x represents the number of terminal groups. All the MXenes are metallic in the absence of surface functionalization with the terminal groups. Due to the functionalization, some MXenes become semiconductors with energy gaps of about 0.25–2.0 eV.^[^ ^61^ ^]^ Owing to high conductivity (> 10^4^ S cm^−1^ for Ti_3_C_2_T*_x_*) given by its very‐low bandgap and surface hydrophilicity, Chertopalov et al. fabricated the Ti_3_C_2_T*_x_*‐based photodetector and evaluation of its feasibility to use for UVC detection. Although the device showed sensitivity to 254‐nm radiation, the photocurrent strongly depended on the ambient atmosphere, where the current signal sharply decayed as the atmosphere changed from Ar to the air. This originated from that the air exposure caused fast oxidation of Ti_3_C_2_T*_x_* to form TiO*_x_*, which deteriorated the conductivity of Mxene film.^[^ ^62^ ^]^ Therefore, to develop Mxene‐based UV detectors, more efforts should focus on improving the stability of Mxene constituents under the oxygen‐rich atmosphere.

##### Wide‐bandgap materials

Wide‐bandgap semiconductors have larger bandgaps than those of widely used semiconductors, such as Si, with values of approximately 2 to 4 eV.^[^ ^63^ ^]^ In general, they exhibit electrical characteristics that are intermediate between those of narrow‐bandgap semiconductors and insulators. The advantage of photodetectors made with wide‐bandgap materials is that they can be made into solar‐blind (or visible‐blind) devices. A solar‐blind photodetector is a detector that does not respond to infrared, visible, and near‐UV light but does respond to UV light with a wavelength of less than approximately 280 nm. A visible‐blind photodetector is a detector that responds to light with a wavelength of less than 400 nm.

Photodetectors employing a narrow‐bandgap material (Si) are typically equipped with a UV bandpass filter, which allows UV light to pass while blocking visible light. In contrast, UV photodetectors that use wide‐bandgap semiconductors are less sensitive to visible light than those using narrow‐bandgap semiconductors; therefore, an additional filter is not required. In addition, UV photodetectors that use wide‐bandgap semiconductors have higher UV/visible rejection rates than those of UV photodetectors that use narrow‐bandgap semiconductors. Therefore, studies of UVC photodetectors using wide‐bandgap materials have been studied more than narrow‐bandgap materials. However, obstacles still exist for wide‐bandgap materials. A major known drawback is the crystal quality. A complex process is essential to manufacture a high‐quality crystal, and thus the cost is not cheap. Low‐quality crystals have increased leakage current due to structural defects. In addition, a relatively large amount of doping is required to secure a large number of carriers due to the high activation energy of the dopant, which reduces carrier mobility. To overcome these problems, numerous studies have been conducted.

###### Zinc oxide

Zinc oxide (ZnO) has a bandgap energy of 3.37 eV, an exciton binding energy of 60 mV, and high thermal ionization at room temperature.^[^ ^64^, ^65^, ^66^ ^]^ ZnO has been applied in various fields, such as UV sensors,^[^ ^67^ ^]^ gas sensors,^[^ ^68^, ^69^ ^]^ and solar cells,^[^ ^70^, ^71^, ^72^ ^]^ owing to its non‐toxicity and excellent optical and electrical properties.^[^ ^73^, ^74^ ^]^ In particular, ZnO is suitable for short‐wavelength devices because of its high exciton energy, which enables effective excitation and emission. It is well suited for optoelectronic devices operating in the UV range.^[^ ^65^, ^75^, ^76^ ^]^ ZnO shows great promise as a UV sensor owing to its fast response^[^ ^77^, ^78^ ^]^ and selective UV detection.^[^ ^79^ ^]^ In addition, ZnO can be manufactured by various deposition methods, such as spin coating,^[^ ^80^, ^81^, ^82^ ^]^ sputtering,^[^ ^83^, ^84^, ^85^ ^]^ and thermal evaporation.^[^ ^86^ ^]^ Furthermore, the optical properties can be controlled through the structural control of the ZnO lattice by doping with appropriate metal ions.^[^ ^65^, ^87^, ^88^ ^]^

**FIGURE 8:**
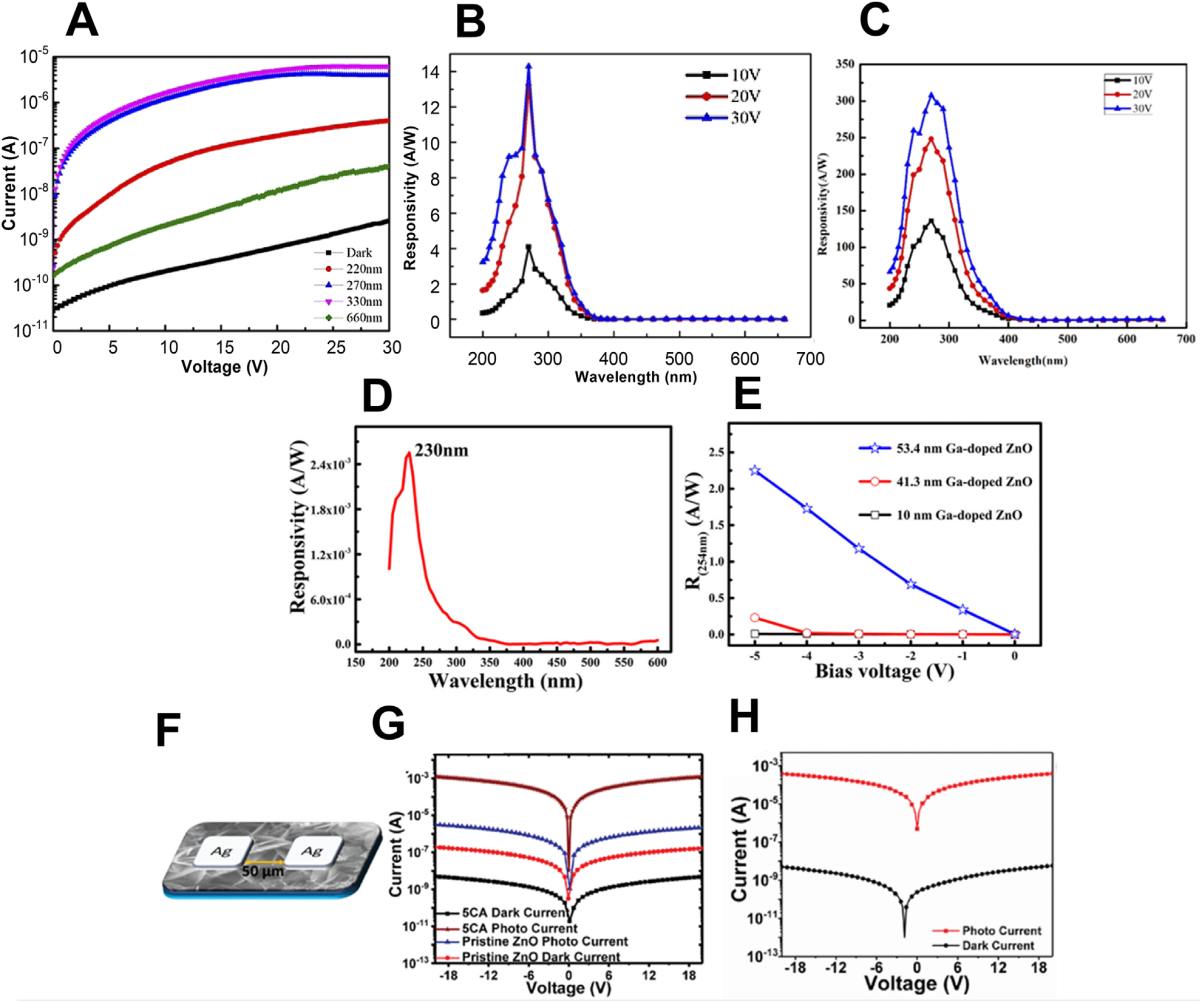
UVC photodetector performance: (A) *I*–*V* curves and (B) responsivities of a 50 nm ZnO/diamond photodetector, (C) responsivities of a 100 nm ZnO/diamond photodetector at different biases. (A–C) Reproduced with permission.^[^ ^94^ ^]^ Copyright 2019, The Optical Society. (D) the responsivity of an Au/MgZnO/Ga‐doped ZnO/In photodetector with a 41.3 nm Ga‐doped ZnO nanolayer without a bias voltage, (E) responsivities of Au/MgZnO/nanolayered Ga‐doped ZnO/In photodetectors with different thickness Ga‐doped ZnO layers under different reverse biases. (D and E) Reproduced with permission.^[^ ^95^ ^]^ Copyright 2019, American Chemical Society. (F) device schematic, (G) *I*–*V* curves of pristine ZnO nanorods and 5 mM Na_3_C_6_H_5_O_7_ samples, and (H) *I*–*V* curves of Pt/5 mM Na_3_C_6_H_5_O_7_ device. (F–H) Reproduced with permission.^[^ ^96^ ^]^ Copyright 2019, IEEE

Various ZnO nanomaterials, such as nanorods,^[^ ^89^, ^90^ ^]^ nanobelts,^[^ ^91^, ^92^ ^]^ and nanoparticles,^[^ ^93^ ^]^ have been used to improve the performance of UV detectors. Nanostructures exhibit efficient photoconduction compared to that of bulk or thin films owing to their large surface‐to‐volume ratio. Therefore, UVC photodetectors using ZnO have been studied extensively over the past few years. For example, Rui et al.^[^ ^94^ ^]^ controlled the thickness of ZnO and deposited it on half of a diamond substrate using a sputtering technique. They fabricated a photodetector by patterning the ZnO and diamond regions of the electrode. The 50 nm ZnO/diamond detector showed a dark current of 0.482 pA and a photocurrent of 3.912 × 10^−6^ A at 30 V under 270 nm illumination, as shown in Figure 8A. In addition, the responsivity of the 50 nm ZnO/diamond photodetector at 10 V under 270 nm illumination was 14.3 A W^−1^, and the photodetector fabricated with a thickness of 100 nm exhibited a responsivity of 308 A W^−1^ (Figure 8B,C).

Although the performance of UVC photodetectors with simple structures has greatly improved, their application in other fields remains difficult. For example, UV light reaching the Earth's surface is greatly attenuated by the atmosphere, requiring high‐performance UVC detectors capable of detecting weak UV light signals. In addition, the high operating voltage of the device is not beneficial for UVC detection. As a result, UV detectors with various structures have been fabricated. For example, Han et al.^[^ ^95^ ^]^ fabricated a UV detector with a metal–insulator–semiconductor (MIS) structure composed of Au/MgZnO/nanolayered Ga‐doped ZnO using pulsed laser deposition. The devices employing Ga‐doped ZnO nanolayers were self‐powered and exhibited a high internal gain in the UVC region. At a bias of 0 V under 230 nm illumination, Ga‐doped ZnO with a thickness of 41.3 nm had a responsivity of 2.55 mA W^−1^, as shown in Figure 8D. As shown in Figure 8E, the 53.4 nm thick sample showed a responsivity of 2.24 A W^−1^ at 5 V under 254 nm illumination.

In addition, UV detectors have been fabricated using various ZnO nanostructures. Agrawal et al.^[^ ^96^ ^]^ fabricated UV detectors using ZnO honeycomb nanostructures via hydrothermal synthesis. They added Na_3_C_6_H_5_O_7_ as a surfactant to obtain different forms of ZnO and a Pt nanoparticle coating to improve the stability and device response speed of ZnO decomposed by UV. Figure 8F shows a schematic of the device. As shown in Figure 8G, the raw ZnO‐based sample exhibited a high dark current of 0.19 µA, a photocurrent of 3.2 µA, and a sensitivity of 15.84 at a bias of 20 V. In addition, the sample with 5 mM Na_3_C_6_H_5_O_7_ achieved a low dark current of 5.17 nA, a photocurrent of 1.26 mA, a sensitivity of 2.4 × 10^5^, a responsivity of 597 A W^−1^, a detectivity of 4.64 × 10^14^ Jones, and an EQE of 2964%. The rise and fall times of the device with 5 mM Na_3_C_6_H_5_O_7_ were 107 and 160 s, respectively. The samples were then coated with Pt nanoparticles to eliminate the effects of UV exposure and stabilize the device. As shown in Figure 8H, the dark current was 5.3 nA, and the photocurrent was 3.13 × 10^−4^ A at a bias of 20 V; the sensitivity and responsivity were 5.9 × 10^4^ and 148 A W^−1^, respectively. Additionally, the device's responsivity improved slightly, reducing the rise time to 90 s, while the fall time remained at 160 s. Under 254 nm irradiation, the switching response of the photocurrent did not change significantly during the cycle, indicating an improvement in the stability of the device under UV radiation.

**FIGURE 9:**
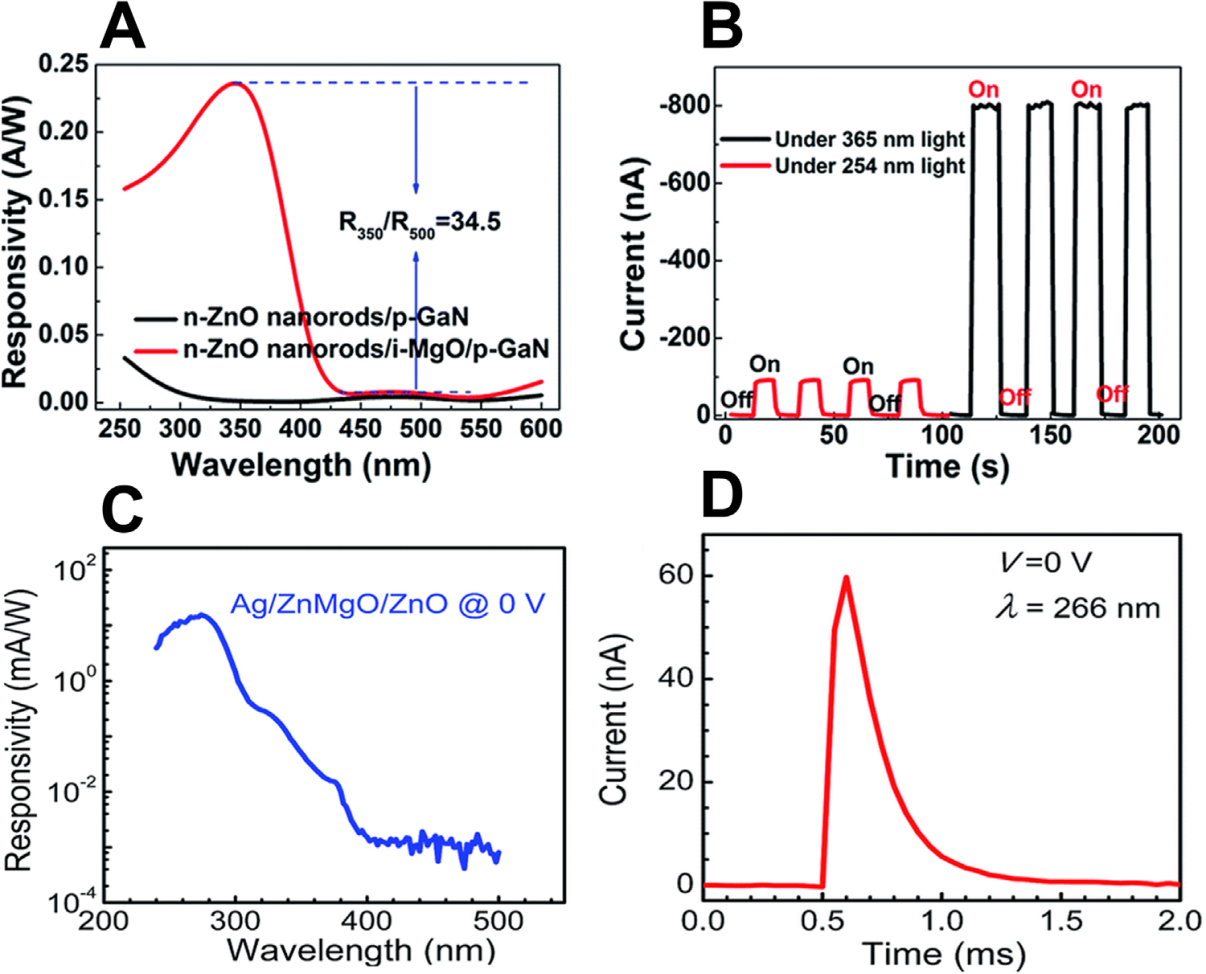
Performance of UVC photodetectors: (A) responsivity and (B) time‐dependent photocurrent of a ZnO nanorods/MgO/GaN photodetector. (A,B) Reproduced with permission.^[^ ^97^ ^]^ Copyright 2015, Royal Society of Chemistry. (C) Responsivity and (D) time‐dependent photocurrent of a Ag/ZnMgO/ZnO photodetector. (C,D) Reproduced with permission.^[^ ^98^ ^]^ Copyright 2017, Royal Society of Chemistry

Moreover, ZnO UVC photodetectors are being actively researched for use in self‐powered devices. For example, Zhou et al.^[^ ^97^ ^]^ developed a self‐powered visible‐blind UVC photodetector with a ZnO nanorod/MgO/GaN structure. Their device achieved a responsivity of 0.16 A W^−1^ under 254 nm illumination at 0 V, and the on/off current values increased from −0.1 to −91 nA, as shown in Figure 9A,B. In addition, low‐cost materials and manufacturing processes are conducive to the further development of different types of devices. Fan et al.^[^ ^98^ ^]^ developed an inexpensive solar‐blind UVC photodetector with a self‐powered Ag/ZnMgO/ZnO vertical structure. As shown in Figure 9C, the fabricated device achieved a responsivity of 16 mA W^−1^ under 275 nm illumination at 0 V. It also showed a rise time of 24 µs and fall time of 300 µs, as shown in Figure 9D.

###### Titanium dioxide

Titanium dioxide (TiO_2_) has been studied more extensively than any other ceramic material because of its non‐toxicity,^[^ ^99^, ^100^ ^]^ natural abundance, thermal stability,^[^ ^101^, ^102^ ^]^ chemical stability,^[^ ^103^, ^104^ ^]^ and hydrophilicity.^[^ ^105^, ^106^ ^]^ TiO_2_ is widely used in many research fields and in many applications, such as the photolysis of pollutants and harmful substances,^[^ ^107^ ^]^ the photolysis of water,^[^ ^108^ ^]^ and artificial photosynthesis.^[^ ^109^ ^]^ TiO_2_ has a bandgap of approximately 3.0 to 3.2 eV and is used as a photocatalyst in various fields.^[^ ^110^, ^111^, ^112^, ^113^ ^]^ Many nanostructures have been developed, such as nanoparticles,^[^ ^114^, ^115^, ^116^ ^]^ nanorods,^[^ ^117^, ^118^, ^119^ ^]^ nanowires,^[^ ^120^, ^121^ ^]^ nanobelts,^[^ ^122^, ^123^ ^]^ and nanotubes.^[^ ^124^, ^125^ ^]^ In addition, research on UVC detectors using TiO_2_ has been actively conducted.

**FIGURE 10:**
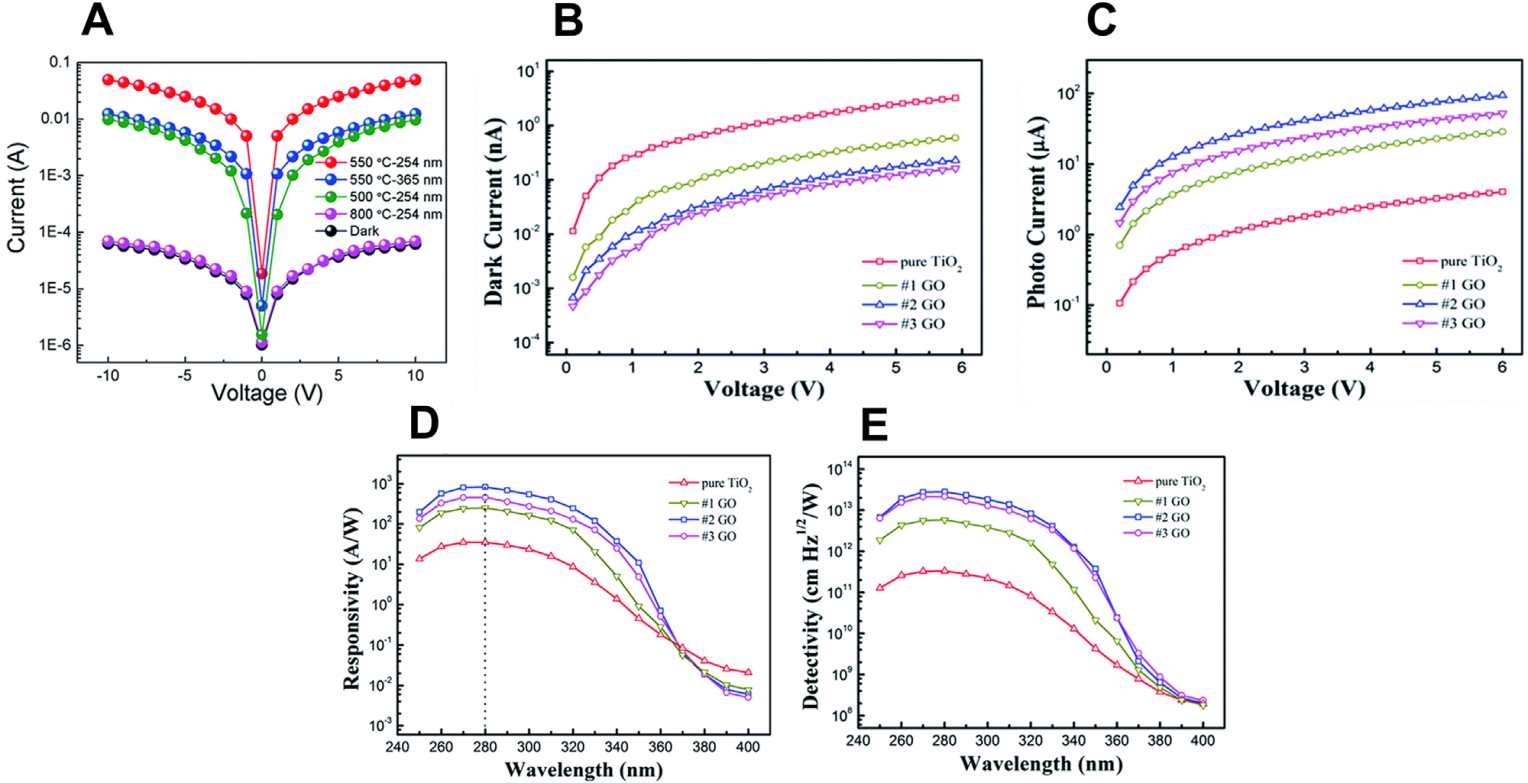
Performance of UVC photodetectors: (A) *I*–*V* curves of a flexible TiO_2_ photodetector. (A) Reproduced with permission.^[^ ^126^ ^]^ Copyright 2017, Royal Society of Chemistry. (B) *I*–*V* curves (dark), (C) *I*–*V* curves (UV illumination), (D) responsivities, and (E) detectivities of a GO device. (B–E) Reproduced with permission.^[^ ^127^ ^]^ Copyright 2015, Royal Society of Chemistry

For example, Wang et al.^[^ ^126^ ^]^ fabricated a transparent and flexible nanoscale UV detector based on TiO_2_ nanowires using an electrospinning method. They deposited TiO_2_ nanowires on flexible substrates. The device achieved a low dark current of approximately pA level and a photocurrent of 47 nA at 10 V under 254 nm illumination (Figure 10A). It also exhibited a high sensitivity of 760 and showed excellent mechanical flexibility and durability.

TiO_2_ has been manufactured as a UVC detector with various structures. For example, Zhang et al.^[^ ^127^ ^]^ improved the performance of metal–semiconductor–metal (MSM)‐structured devices by tuning the Schottky contact between TiO_2_ and Au electrodes using graphene oxide (GO), which has excellent electrical conductivity. The prepared sample was deposited by spin‐coating GO at 2000–6000 rpm, and interdigital shapes were fabricated using lithography techniques. The GO films fabricated at 6000 (#1), 4000 (#2), and 2000 (#3) rpm showed dark currents of 0.59 (±0.03), 0.23 (±0.02), and 0.16 (±0.03) nA, respectively, at a voltage of 6 V, as shown in Figure 10B. In addition, the 6000 and 4000 rpm samples showed photocurrents of 28.7 (±0.1) and 94.3 (±0.1) µA, respectively, under 280 nm irradiation at 6 V, as shown in Figure 10C. As shown in Figure 10D,E, the 4000 rpm sample achieved a responsivity of 826.8 A W^−1^ and a detectivity of 2.82 × 10^13^ Jones under 280 nm irradiation at 6 V.

###### Gallium oxide

Gallium oxide (Ga_2_O_3_) is a semiconductor with a bandgap of 4.7 to 4.9 eV^[^ ^128^, ^129^, ^130^ ^]^ and good chemical and thermal stabilities.^[^ ^131^, ^132^, ^133^ ^]^ The wide bandgap of Ga_2_O_3_ provides an absorption cutoff edge at approximately 280 nm, which is useful for UVC detection. In addition, Ga_2_O_3_ can be manufactured at a low cost. Ga_2_O_3_ has five isomers.^[^ ^134^ ^]^ Among them, β‐Ga_2_O_3_ has a bandgap of approximately 4.7 to 4.9 eV at room temperature, and it is assumed to be stable compared to other isomers.^[^ ^135^, ^136^ ^]^ Considerable research has been conducted on Ga_2_O_3_‐based UV detectors.

**FIGURE 11:**
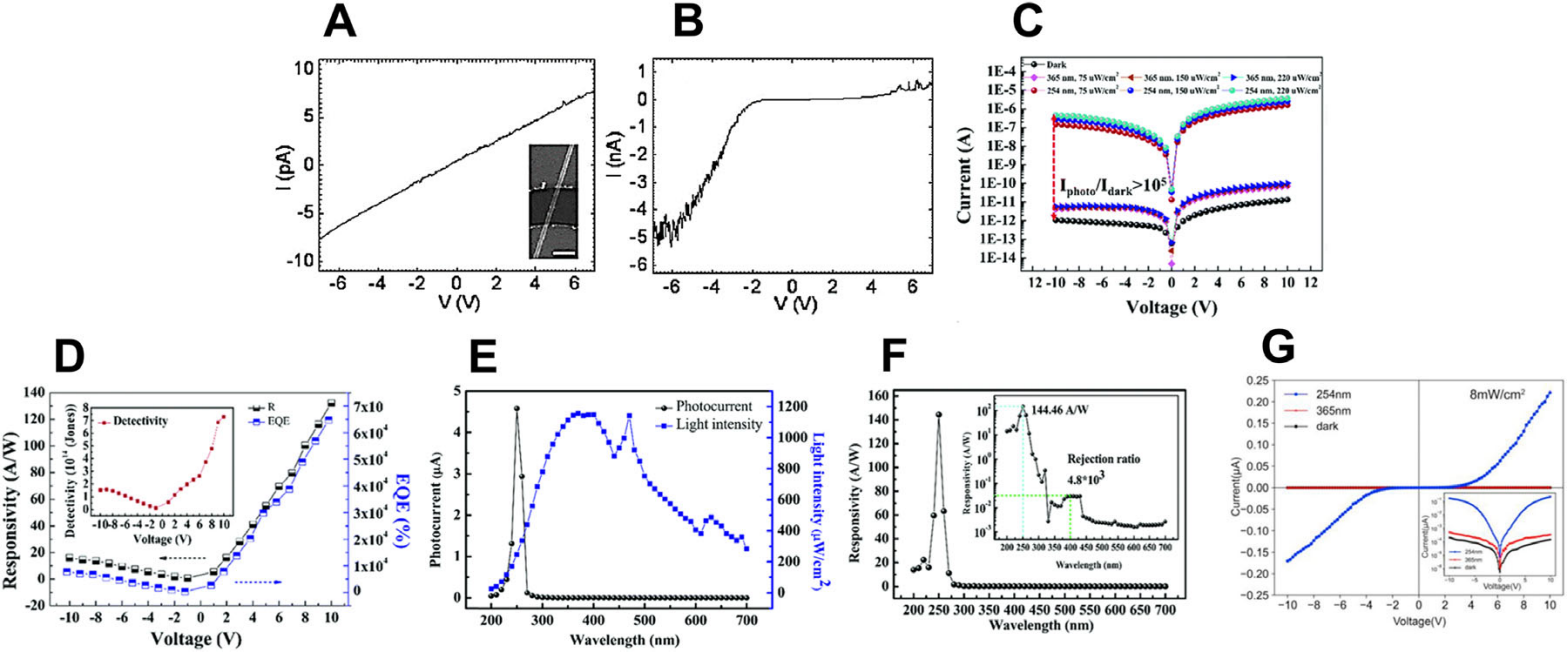
UVC photodetector performance: (A) *I*–*V* curve (dark) and (B) *I*–*V* curve (under 254 nm). (A,B) Reproduced with permission.^[^ ^137^ ^]^ Copyright 2006, American Institute of Physics. (C) *I*–*V* curves, (D) EQE and responsivity, (E) photocurrent and light intensities, and (F) photoresponse spectrum of a Ga_2_O_3_ Schottky diode UVC photodetector. (C–F) Reproduced with permission.^[^ ^138^ ^]^ Copyright 2019, Royal Society of Chemistry. (G) *I*–*V* curves of a GaN/β‐Ga_2_O_3_ nanowire heterojunction. (G) Reproduced with permission.^[^ ^139^ ^]^ Copyright 2021, Elsevier B.V.

Feng et al.^[^ ^137^ ^]^ fabricated a photoconductor structure by depositing Ga_2_O_3_ nanowires on an Au‐coated Si substrate using an evaporation method. As shown in Figure 11A,B, the device achieved a dark current of approximately 10^−12^ A and exhibited a photocurrent that was approximately three times higher under 254 nm illumination. In addition, the rise/fall times under 254 nm illumination at a bias of −8 V were 0.45 and 0.09 s, respectively. Moreover, the photocurrent curve of the device was asymmetric and nonlinear. This behavior was due to the poor ohmic contact between the electrode and the nanowire.

Ga_2_O_3_‐based UV detectors with various structures have been proposed to overcome the disadvantages of photoconductor‐type UV detectors. Ga_2_O_3_ thin films can generally be deposited by radio frequency (RF) magnetron sputtering, metal oxide chemical vapor deposition (MOCVD), and molecular beam epitaxy (MBE).

RF sputtering can be applied to rapidly fabricate high‐quality coatings for UV detectors using simple equipment. For example, Liu et al.^[^ ^138^ ^]^ fabricated a β‐Ga_2_O_3_ Schottky diode UV detector on a sapphire substrate by RF sputtering. Their device exhibited a very low dark current of 1.32 × 10^−11^ A and a sensitivity of 2.83 × 10^5^ at 10 V under 254 nm illumination (Figure 11C). As shown in Figure 11D, the highest responsivity was 132.3 A W^−1^ at an applied voltage of 10 V. In addition, a maximum photocurrent of 4.58 × 10^−6^ A and a responsivity of 144.46 A W^−1^ were achieved at a bias of 10 V under 250 nm illumination, as shown in Figure 11E,F, respectively. Jia et al.^[^ ^140^ ^]^ fabricated a NiO/β‐Ga_2_O_3_ heterojunction‐based UV detector in which NiO and β‐Ga_2_O_3_ were deposited on a sapphire substrate by RF sputtering. Heterojunction structures with proper band alignment can facilitate charge separation and collection. Their device achieved a responsivity of 27.43 A W^−1^ under 254 nm at 10 V and a detectivity of 3.14  ×  10^12^ Jones (−10 V).

MOCVD is a CVD technique that uses the pyrolysis reaction of organometallics to perform vapor‐phase epitaxial growth of thin films.^[^ ^141^ ^]^ Nanostructures can be grown in a vacuum using the MOCVD process.^[^ ^142^ ^]^ Ding et al.^[^ ^139^ ^]^ fabricated a UV detector with a GaN/β‐Ga_2_O_3_ nanowire heterojunction structure by MOCVD. Their device achieved a dark current of 1.6 × 10^−10^ A and a photocurrent of 0.22 µA, as shown in Figure 11G. It also exhibited a sensitivity of 1.375 × 10^3^, a detectivity of 1.2 × 10^11^ Jones, and a responsivity of 27.5 mA W^−1^ under 254 nm irradiation at 10 V.

MBE can improve the performance of UV detectors because the deposited thin film becomes more uniform over time. Pratiyush et al.^[^ ^143^ ^]^ fabricated a β‐Ga_2_O_3_‐based self‐powered UV detector using MBE. It had an MSM structure in which Au and Ti were formed into metal contacts using an e‐beam evaporator on a Ga_2_O_3_ thin film. Their device achieved a dark current and photocurrent of 1.7 nA and 19 µA, respectively, under 255 nm irradiation at −15 V (reverse bias). In addition, they achieved a responsivity of 1.4 mA W^−1^ and an EQE of 0.5% under 255 nm irradiation at 0 V, indicating that their device was self‐powered. Furthermore, the rise/fall response times were confirmed to be 1.1 and 0.3 s, respectively.

**FIGURE 12:**
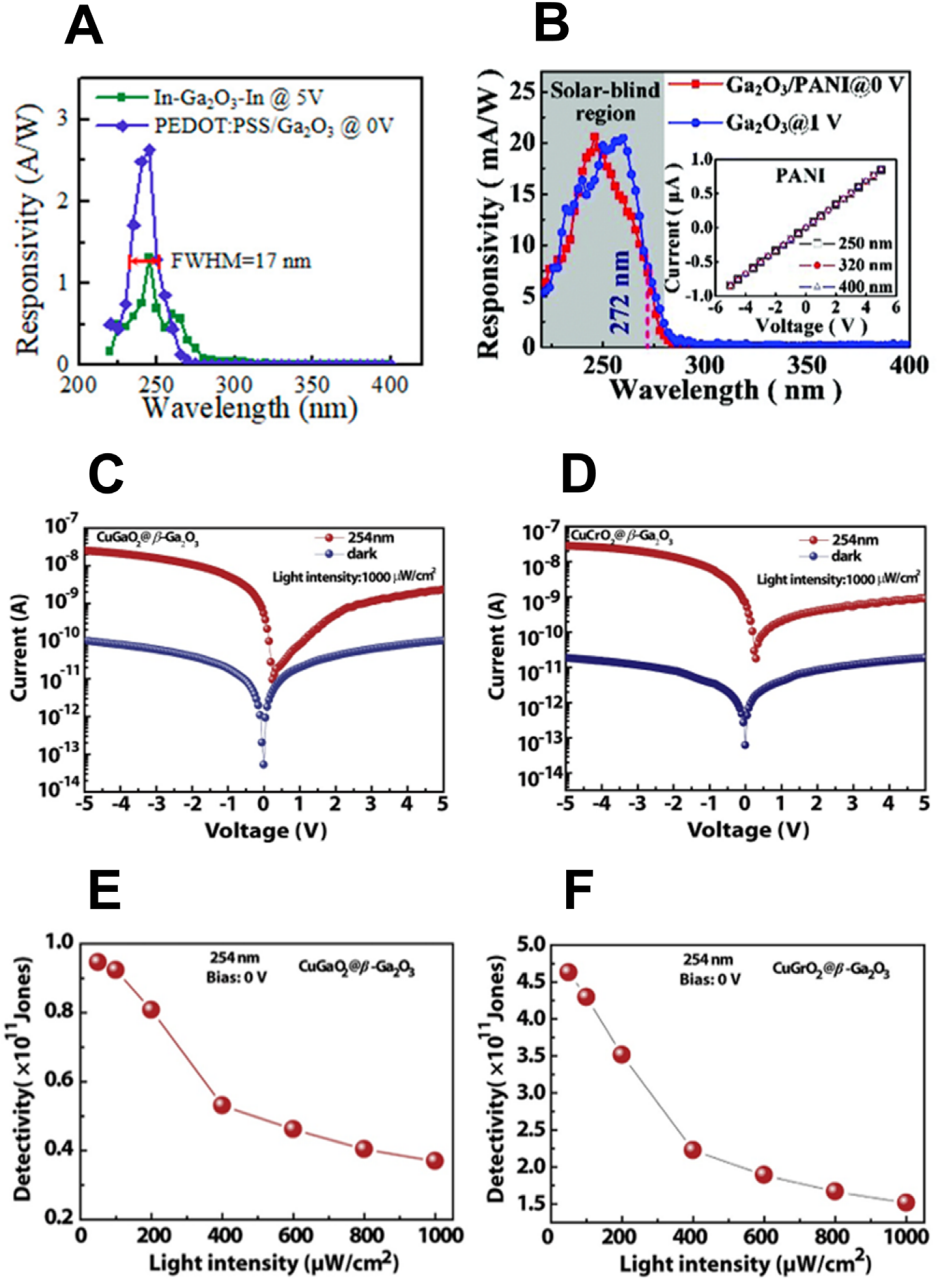
UVC photodetector performance: responsivities of (A) a PEDOT:PSS/Ga_2_O_3_ p–n junction. (A) Reproduced with permission.^[^ ^144^ ^]^ Copyright 2019, American Chemical Society. (B) a Ga_2_O_3_ microwire/polyaniline heterojunction. (B) Reproduced with permission.^[^ ^145^ ^]^ Copyright 2020, Royal Society of Chemistry. *I*–*V* curves of (C) CuGaO_2_/β‐Ga_2_O_3_ and (D) CuCrO_2_/β‐Ga_2_O_3_; and responsivities of (E) CuGaO_2_/β‐Ga_2_O_3_ and (F) CuCrO_2_/β‐Ga_2_O_3_. (C–F) Reproduced with permission.^[^ ^146^ ^]^ Copyright 2021, Elsevier B.V.

Numerous studies have been conducted on self‐powered devices. Ga_2_O_3_ has a wide bandgap and is generally fabricated with p–n junctions and heterojunctions. For example, Wang et al.^[^ ^144^ ^]^ fabricated a self‐powered solar‐blind UVC photodetector with an organic/inorganic p–n junction structure based on poly(3,4‐ethylenedioxythiophene): poly(styrenesulfonate) (PEDOT:PSS) and β‐Ga_2_O_3_. They fabricated Ga_2_O_3_ microwires through a CVD process and used PEDOT:PSS as the hole‐transport layer. As shown in Figure 12A, a responsivity of 2.6 A W^−1^ and a high rejection rate (*R*_245nm_/*R*_400nm_ = 9 × 10^4^, *R*_245nm_/*R*_280nm_ = 1 × 10^3^) were achieved at 0 V. Similarly, Wang et al.^[^ ^145^ ^]^ fabricated a self‐powered solar‐blind photodetector based on a β‐Ga_2_O_3_ microwire/polyaniline heterojunction. A centimeter‐sized β‐Ga_2_O_3_ microwire was fabricated using a CVD process. Their UVC detector had a responsivity of 21 mA W^−1^ and a rejection ratio (*R*_246nm_/*R*_400nm_) of 3 × 10^2^ at 0 V, as shown in Figure 12B.

Wu et al.^[^ ^146^ ^]^ fabricated a stable self‐powered UVC photodetector using a simple solution process. The device comprised a p–n heterojunction based on n‐β‐Ga_2_O_3_/p‐CuMO_2_ (M: Ga^3+^, Cr^3+^). As shown in Figure 12C, the CuGaO_2_/β‐Ga_2_O_3_ p–n photodetector showed a dark current of 51 fA at 0 V and a photocurrent of 0.54 nA under 254 nm illumination. The CuCrO_2_/β‐Ga_2_O_3_ detector showed a dark current of 61 fA and a photocurrent of 0.70 nA, as shown in Figure 12D. In addition, the photodetectors achieved detectivities of 0.9 × 10^11^/4.7 × 10^11^ Jones and rejection ratios (*R*_254nm_/*R*_365nm_) of 2 × 10^4^/2.8 × 10^4^, respectively, as shown in Figure 12E,F.

UVC photodetectors based on wide‐bandgap semiconductors are generally manufactured as solar‐blind (or visible‐blind) devices. Without a separate UV pass filter, the sensitivity to visible light is low, and thus the UV/visible rejection rate is high. It is possible to manufacture self‐powered UVC detectors with p–n junctions and heterojunctions. Wide‐bandgap detectors show excellent performance in the solar‐blind region and are promising candidates for UVC photodetectors. The merit parameters of the recent UVC detectors using various wide‐bandgap photoactive materials were summarized in Table 2.

**TABLE 2:** Performance of recent UVC photodetectors based on wide‐bandgap materials

Photo‐absorber	Device structure	Wavelength (nm)	Voltage (V)	R (mA W^−1^)	D (Jones)	Rise/fall time	Rejection ratio	Flexible device	Ref.
β‐Ga_2_O_3_	MSM	265	0	11.6 × 10^−3^		16.6/35.2 s		Yes	[147]
C‐ZnO QDs	MSM	224	5	88.4		<85 ms	I_224_:I_300_ ≈ 11	Yes	[20]
β‐Ga_2_O_3_/NiO	PN	245	10	27.4 × 10^3^	3.1 × 10^12^				[140]
Graphene QDs	Schottky	254	5	2.1	9.59 × 10^11^	64/43 ms	Blind to 365 nm and white light		[148]
ZnO–Ga_2_O_3_ core–shell	Heterojunction	251	0	9.7	6.29 × 10^12^	0.1/0.9 ms	R_251_:R_400_ ≈ 6.9 × 10^2^		[149]
GaN/Sn:Ga_2_O_3_	PN	254	0	3.05 × 10^3^	1.69 × 10^13^	180 ms	R_254_:R_400_ ≈ 5.9 × 10^3^		[150]
GaN/Ga_2_O_3_	PN	254	0	28.44	6.17 × 10^10^	140/70 ms			[28]
PEDOT:PSS/Ga_2_O_3_	Heterojunction	254	0	37.4	9.2 × 10^12^	3.3/71.2 µs	R_250_:R_360_ ≈ 7 × 10^3^		[27]
Polyaniline/MgZnO	PN	250	0	0.16	1.5 × 10^11^	< 0.3 ms	R_250_:R_400_ ≈ 10^4^		[151]
Ag/ZnMgO/ZnO	Schottky and heterojunction	275	0	16	5 × 10^9^	24/300 µs	R_275_:R_400_ ≈ 10^4^		[98]
Nb:SrTiO_3_/Ga_2_O_3_	Heterojunction	254	0	2.6		70/210 ms			[152]
Diamond/β‐Ga_2_O_3_	Heterojunction	244	0	0.2	6.9 × 10^9^		R_244_:R_400_ ≈ 1.4 × 10^2^		[153]
TiO_2_–PANI/TiO_2_	PN and heterojunction	254	0				I_254_:I_365_ ≈ 1:2		[154]
TiO_2_ nanopaper	Heterojunction	254	1					Yes	[126]
TiO_2_/diamond	MSM	220	30	130		1 ms	R_220_:R_400_ ≈ 68:1		[155]
TFB/CaSnO_3_/SnO_2_	PIN	254	0	2.25	1.56 × 10^10^	80/70 ms	I_254_:I_365_ ≈ 5.5		[156]
SnO_2_ nanowires	Photoconductor	250	3	1.2 × 10^10^					[157]

###### III‐nitride

The properties of group III‐nitride have a wide‐bandgap, high carrier mobility, and excellent chemical stability.^[^ ^158^ ^]^ The III‐nitride semiconductors include GaN, AlN, InN, and multi‐element alloys (AlGaN, InGaN, and AlGaInN), and adjust bandgap energy (3.4–6.2 eV) by changing the composition of the alloy.^[^ ^159^, ^160^, ^161^, ^162^ ^]^ These advantages enable the manufacture of a solar‐blind UV detector using III‐nitrides.

Wang et al.^[^ ^163^ ^]^ fabricated a GaN‐based self‐powered UVC photodetector by MOCVD. Their device was constructed with an asymmetric MSM structure with the addition of AlGaN. The device composed of Al_0.1_Ga_0.9_N/GaN showed high responsivity of 0.005A/W at 0V 240 nm irradiation and 13.56A/W at 3V. It also achieved 97.95 rejection ratios (R_240nm_/R_390nm_) at 0V. Zhuo et al.^[^ ^164^ ^]^ fabricated a self‐powered UVC photodetector based on a MoS_2_/GaN p‐n heterojunction. Their device exhibited a dark current of 3 × 10^–12^ A, a photocurrent of 2.7 × 10^–7^ A at 0 V under 265 nm illumination with an intensity of 2.4 mW cm^–2^. The MoS_2_/GaN p‐n photodetector exhibited a high responsivity of 187 mA W^−1^ and a detectivity of 2.34 × 10^13^ Jones at 265 nm illumination with an intensity of 1 µW cm^−2^. Furthermore, the rise/fall times were 0.302 ms/3.605 ms (100 Hz) and 46.4 µs/114.1 µs (5 kHz) for 265 nm illumination with an intensity of 2.4 mW cm^−2^, respectively. Kaushik et al.^[^ ^165^ ^]^ treated the AlN surface with meso‐5,10,15‐triphenyl‐20‐(*p*‐hydroxyphenyl)porphyrin Zn(II) complex (ZnTPP(OH)) to form a self‐assembled monolayer and fabricated a photodetector. Their device was fabricated with an MSM structure using Ni. At 200 nm illumination, the Ni/treated AlN/Ni device exhibited a dark current of 1.46 × 10^–11^ A, achieving a dark current reduction of about 10 times compared to that of Ni/bare AlN/Ni. Also, at −5V, Ni/treated AlN/Ni and Ni/bare AlN/Ni showed dark currents of 2.41 × 10^–12^ A and 9.24 × 10^–11^ A, respectively. In addition, the responsivity of Ni/treated AlN/Ni and Ni/bare AlN/Ni at 5V was 0.6 mA/W and 0.3 mA/W, respectively.

###### Silicon carbide

Silicon carbide (SiC) is a promising material for high‐temperature semiconductors because it has excellent heat resistance, corrosion resistance, oxidation resistance, and heat resistance.^[^ ^166^ ^]^ Due to excellent material characteristics such as a wide energy band gap, high breakdown voltage, high thermal conductivity, and saturation movement speed, it has been spotlighted in the application as a power device, and in particular, it is widely used in applications as a high voltage and high‐temperature device.^[^ ^167^ ^]^ Methods for manufacturing SiC, which are widely used as a high‐temperature semiconductor and photoelectric device materials, include a chemical vapor deposition method, a PECVD method, a sputtering method, and a photo‐CVD method.^[^ ^168^, ^169^, ^170^, ^171^ ^]^ SiC is a compound semiconductor composed of a covalent bond between a Si atom and a C atom. In general, it has 3C‐SiC, 4H‐SiC, 6H‐SiC, etc. Chang et al.^[^ ^172^ ^]^ fabricated a photodetector by depositing a cubic SiCN film on a Si substrate using Rapid Thermal Chemical Vapor Deposition. Their device was fabricated with the MSM structure. The SiCN photodetector shows selective detection that responds only to the illumination of 254 nm under irradiation of various illuminations (254 nm, 366 nm, 480 nm). The SiCN detector at room temperature achieved a sensitivity of 6.5 at −5V. Yu et al.^[^ ^173^ ^]^ fabricated a self‐powered β‐Ga_2_O_3_/4H–SiC pn heterojunction UVC photodetector using the pulse laser deposition method. Their device exhibited a dark current of 6.22 × 10^−12^ A, a photocurrent of 1.03 × 10^−8^ A, and a rise/fall time of 11 ms/19 ms at 0V. Besides, a responsivity of 10.35 mA/W, a detectivity of 8.8 × 10^9^ Jones, and an EQE of 4.77% were achieved.

## 4 ASPECTS OF FLEXIBLE UVC PHOTODETECTOR

### 4.1 Flexible substrates

Quartz is the most commonly used (and most transparent) substrate for fabricating UVC photodetectors owing to its high transmittance in the deep‐UV region, up to ∼90% despite its great thickness. Therefore, quartz substrates allow a large quantity of photons to reach the active layer, thus remarkably boosting the responsivity of UVC photodetectors compared to those constructed on commercial glass. Unfortunately, the rigidity of this substrate limits its use for many practical applications because there is a high demand for wearable and flexible UVC photodetectors.

To date, several polymer materials, such as PET, PS, PI, and PEN, have been used to study flexible optoelectronic devices because of their inherent mechanical strength and thermal stability. However, a critical disadvantage of utilizing these polymer substrates for deep‐UV detectors is that UVC irradiation cannot penetrate these substrates into the active layer. From this point of view, the incident directions of UV illumination on the device are important issues to be discussed.

#### 4.1.1 Top‐to‐bottom illumination

**FIGURE 13:**
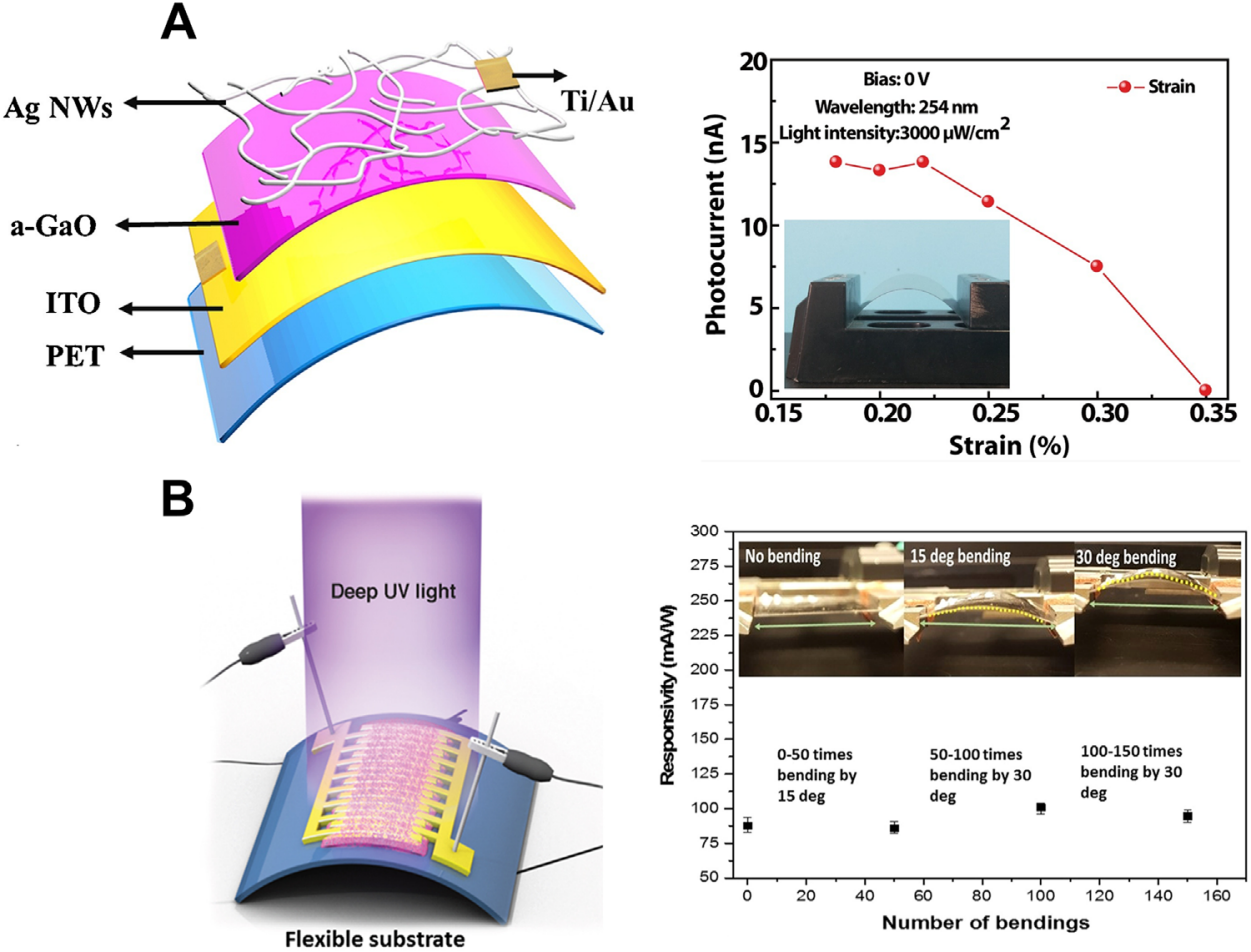
(A) Strain‐dependent photocurrent of a flexible deep‐UV photodetector based on Ag nanowires. (A) Reproduced with permission.^[^ ^19^ ^]^ Copyright 2021, Elsevier B.V. (B) Highly stable and reproducible carbon‐doped ZnO QD‐based photodetector. (B) Reproduced with permission.^[^ ^20^ ^]^ Copyright 2018, Elsevier B.V.

Top‐illuminated device structures are typically developed in two models. In the first model, a photodetector is a built‐in vertical structure in which UVC radiation can pass through the top electrode to reach the active layer. As a result, conductive PEDOT:PSS layers, graphene monolayers, metal grids, and metal nanowires are potential candidates for the top layer. For example, Wang et al. constructed a self‐powered UVC wearable photodetector based on silver nanowires (Figure 13A).^[^ ^19^ ^]^ The device showed a good detectivity of 3.1 × 10^9^ Jones under 254 nm illumination along with a high R_254_/R_365_ ratio of 70 and good mechanical stability, even under 0.25% strain.

In the second model, researchers have exploited lateral architectures involving conducting electrode layers with unique patterns. In this case, the shadow mask technique must be strictly controlled because the distance between the electrodes can affect the transit length of the charge carriers. For instance, Mitra et al.^[^ ^20^ ^]^ designed a flexible UVC photodetector using a serpentine pattern of Ti electrodes with a distance of as close as 20 µm between interdigitated Ti electrodes. It was highly responsive, reproducible, and immune to stress (Figure 13B).

#### 4.1.2 Bottom‐to‐top illumination

**FIGURE 14:**
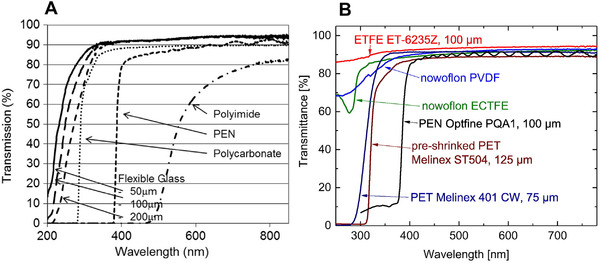
Wavelength‐dependent transmittance of various substrates. (A) Reproduced with permission.^[^ ^176^ ^]^ Copyright 2014, Springer‐Verlag Berlin Heidelberg. (B) Reproduced with permission.^[^ ^177^ ^]^ Copyright 2016, Elsevier B.V.

None of the above‐mentioned flexible substrates can be utilized to construct bottom‐illuminated device structures because they have restricted transmittance in the UVC region. Work to overcome the selective transmission of UVC light is ongoing in several research groups. Interestingly, Willow Glass™ by Corning exhibits not only flexibility but also good optical transmission in the deep‐UV region. Moreover, the transparent glasses take over the plastic substrates in significantly high thermal stability (700°C). This allows the high‐temperature processes to improve the crystallinity of photoactive material as well as material‐electrode contact for facilitating charged carrier transfer and collection.^[^ ^174^ ^]^ Another benefit of the flexible glass substrate is its optical durability. The Willow Glass will not yellow or darken upon UV exposure as other polymer films will.^[^ ^175^ ^]^ The thickness‐dependent transmission of this type of flexible glass is illustrated in Figure 14A. As shown, 100 µm thick glass has a cut‐off wavelength of 250 nm (approximately 50% transmittance).^[^ ^176^ ^]^ In addition, some polymer films with an appropriate thickness, which provides sufficient mechanical tolerance and UVC transparency, are potential candidates for flexible substrates for UVC detectors. Figure 14B shows the UV–vis transmittance spectra of various polymers, including ethylene tetra fluoro ethylene (ETFE), Nowoflon, polyvinylidene fluoride, ethylene chlorotrifluoroethylene, PEN, and PET.^[^ ^177^ ^]^ Among them, ETFE films (a copolymer of ethylene and perfluoroethylene) show a high transmittance (∼85%) at 250 nm, making it a promising material for the upcoming generation of flexible UVC photodetectors.

On the other hand, most device contacts of flexible devices require deposition processes such as photolithography, sputtering, electron beam, and thermal evaporation for patterning, and are manufactured based on metals such as Au.^[^ ^178^, ^179^, ^180^, ^181^ ^]^ In general, it is manufactured by a thermal evaporation process using a shadow mask, which is relatively expensive and time‐consuming. In addition, this method has disadvantages in terms of cost, and compatibility with flexible substrates may be limited. To overcome these limiting aspects, solution‐based contact development is being promoted. Liu et al.^[^ ^182^ ^]^ manufactured a high‐conductivity film of graphene/poly(3,4‐ethylenedioxythiophene):poly(styrenesulfonate) (PEDOT:PSS) (PH1000) through spray coating. Their device was fabricated with a composition of graphene/PH1000/PEDOT:PSS/P3HT:PCBM/Al, where graphene/PH1000 film was used as the bottom electrode. They investigated the mechanical stretchability of the film, and there was no significant change in resistance when the graphene/PH1000 film was stretched from 0% to 20% for 50 cycles and then restored to a flat state. In addition, they conducted a bending test of the graphene/PH1000 film. As a result, the resistance of the graphene/PH1000 film does not change significantly during bending 1000 times with a radius of 5 mm. Harper et al.^[^ ^183^ ^]^ reported an aerosol spray laser lithography process using the aerosol spray for deposition and laser toner for patterning. Their proposed aerosol spray laser lithography process can perform patterning of various sizes and shapes without thermal evaporation. In addition, it can be applied to various substrates, including flexible substrates, with low cost and process conditions of less than 155°C.

### 4.2 Low‐dimensional photoactive materials

In addition to the apposite substrate and conductive electrode, another vital aspect needing to discuss in flexible photodetector is the nanostructure dimension of photoactive materials, including zero‐dimensional (0D), one‐dimensional (1D), and two‐dimensional (2D) semiconductors. As compared to the bulk, the nanostructure dimension exhibits not only appealing optoelectrical properties but also outstanding mechanical flexibility.

First, 0D materials are tiny‐sized particles having diameters less than 100 nm. For UVC detection, the diameters of particles are usually reduced to the range from several to 20 nm, called quantum dots (QDs). Owing to the quantum confinement effect (i.e., size‐dependent bandgap of QDs), the absorption of photoactive materials can be tuned by varying the size, where the absorption peak tends to blue‐shift as reducing particle size.^[^ ^156^ ^]^ Xia et al. reported a solution‐processed solar‐blind UVC detector based on ZnS QDs. The UV‐Vis spectrum of 2‐nm‐diameter spherical ZnS QDs showing a cut‐off wavelength less than 300 nm indicated a high selectivity to UVC radiation without the interference of light in other spectra. However, ZnS QDs‐device showed low responsivity of 0.1 mA W^−1^ even though the high required external bias of 40 V.^[^ ^12^ ^]^ The reason is that ZnS QDs has poor charge transport issue because the photogenerated charges have to travel through the adjacent QDs, where they get trapped before reaching the electrodes. The inefficient transfer of charged carriers among the adjacent QDs would be aggravated by increasing physical distance as deformation of the substrate.^[^ ^184^ ^]^ This is probably the main reason why QDs‐based flexible UVC photodetector has rarely been studied up to now.

To overcome the distance between individual units of photoactive QDs, 1D and 2D semiconductors have been gaining much attention to studying photodetectors in recent years. Opposed to QDs‐based photodetector, the heterojunction of ZnS/InP nanowires built onto a PET substrate exhibited excellent photocurrent stability under different bending states (at bending angles of 0–120^°^).^[^ ^185^ ^]^ For another example, wide‐bandgap UVC photo‐absorber mats consisting of Zn_2_GeO_4_ and In_2_Ge_2_O_7_ nanowire crisscrossing network were fabricated by CVD method, followed by transferring onto flexible adhesive PET tape substrate as reported by Liu et al.^[^ ^186^ ^]^ The device showed the nearly unchanged *I*–*V* curve under 254 nm illumination after 100 cycles of bending, suggesting outstanding stability and reproducibility of the flexible devices. On the other hand, 2D materials are more compatible with current micromanufacturing techniques and are more conveniently fabricated as compared to 1D materials. Hu et al. fabricated a 0.75‐nm‐thick GaS nanosheet‐based photodetector on a PET substrate and demonstrated its mechanical stability by evaluating the photoresponse of the device upon 254 nm illumination under both different bending angles and before/after bending states. Although the photocurrent decreased with the bending angles, the photocurrent was retained constantly at the beginning and after bending 20 times. This phenomenon reflected that the device was durable under deformation but its performance was dependent on bending angles, which determined the illuminated active area.^[^ ^187^ ^]^

## 5 CONCLUSION AND OUTLOOK

In this review, we introduce and discuss strategies for fabricating self‐powered UVC photodetectors on flexible substrates in terms of the structure, material, and direction of incoming radiation.

First, considering the feasibility of self‐powered features, photodetectors constructed with selective heterojunctions, such as p–n and p–i–n structures, generally operate without any energy consumption owing to built‐in potentials at the interfaces of the constituents. In contrast, most photoconductors and symmetric MSM photodetectors do not exhibit self‐powered operation but possess high responsivity along with simple fabrication. Owing to their ability to be self‐powered and simple fabrication procedures, photodetectors with asymmetric MSM and Schottky structures have attracted considerable attention in recent research.

Second, regarding photoactive regions, numerous narrow‐ and wide‐bandgap semiconductors have been utilized in the vital role of core photo‐absorbers in photodetectors. From the standpoint of detection performance (e.g., responsivity, detectivity, and response speed), narrow‐bandgap materials are favorable for optical‐filter‐assisted photodetectors owing to their intrinsically high carrier mobility and sensitivity to high‐energy photons. In contrast, despite their inferior performance, deep‐UV photodetectors based on wide‐bandgap photo‐absorbers exhibit solar‐blind (or visible‐ and infrared‐blind) properties accompanied by higher stability during storage under ambient conditions.

To enhance performance and facilitate practical application, recent studies have focused on two primary strategies: (i) developing down‐conversion materials, which are expected to replace complicated optical filters, for direct integration into devices based on narrow‐bandgap materials; (ii) improving the carrier mobility of wide‐bandgap photo‐absorbers by doping, incorporating graphene‐related materials (e.g., graphene oxide, graphene quantum dots), and inserting appropriate interfacial/carrier‐transfer layers.

Third, in addition to device performance, flexibility has a decisive impact on the practical applications of wearable electronics. Diverse polymer films, such as PET, PS, PI, and PEN, serve as low‐cost and highly durable substrates for effective flexible photodetectors. However, their usage is limited by low‐temperature procedures, employing solution processes below 500°C. Another disadvantage of these types of plastic substrates is that they only operate on one side with top‐illuminated light owing to their low transmittance in the UVC spectra. Consequently, the constituents of photodetectors on highly transparent plastic substrates, such as ETFE and flexible glass, have been the focus of the next generation of lightweight and wearable deep‐UV detectors.

In summary, although UVC detection is still in its infancy compared to the detection of UVA and other photon spectra (relatively low output signal and feasibility in practical applications), recent research has studied different key components, including configurations, materials, and substrates, to acquire battery‐free, super‐sensitive, ultra‐stable, ultra‐small, and portable UVC photodetectors. Hence, they have the potential for numerous applications in the future, including facilitating the next era “Internet of things” for communication and civil systems, integration into skin‐attached electronics, and utilization in spacecraft.

## CONFLICT OF INTEREST

The authors declare no conflict of interest.
